# Polymer-Based p‑Type Exciplex Systems for Air-Stable and Processable Organic Persistent Luminescence

**DOI:** 10.1021/jacsau.6c00384

**Published:** 2026-05-26

**Authors:** Feilong Liu, Liyi Li, Angelo Homayoun All, Jonathan W.C. Wong, Jean-Claude G. Bünzli

**Affiliations:** † Research Center for Eco-environmental Engineering, 74549 Dongguan University of Technology, Dongguan 523808, China; ‡ Department of Applied Biology and Chemical Technology, 26680 The Hong Kong Polytechnic University, Hong Kong SAR 999077, China; § Department of Chemistry, 26679 Hong Kong Baptist University, Hong Kong SAR 999077, China; ∥ Institute of Chemical Sciences & Engineering, Swiss Federal Institute of Technology, Lausanne 1015, Switzerland

**Keywords:** long persistence luminescence, exciplex, polymer, p-type

## Abstract

We report the first p-type polymer-based multicomponent exciplex system exhibiting persistent luminescence (PersL). Using poly­(*N*-vinylcarbazole) as the polymer donor host, with 2,4,6-*tris*(4-methoxyphenyl)­pyrylium tetrafluoroborate and *N*,*N*′-*Bis*(1-naphthalenyl)-*N*,*N*′-*bis*-phenyl-(1,1′-biphenyl)-4,4′-diamine as the acceptor and trap material, respectively, this system achieves an afterglow lasting over 3 h. It directly addresses the critical drawbacks of existing n-type or small-molecule exciplex systems, namely rapid quenching of their PersL by oxygen and poor processability. Moreover, the developed polymer-based p-type exciplex is readily prepared through a one-pot “boil-up” protocol followed by a brief annealing step, and presents excellent thermal moldability. Through detailed charge carrier dynamics investigations and the study of the effects of various environmental conditions, we uncover new mechanistic insights and their underlying phenomena. This work establishes a robust and process-friendly platform for organic PersL, potentially broadening its applications in anticounterfeiting—as demonstrated here with a multilayer anticounterfeiting QR-code, data encryption, and wearable photonic technologies.

## Graphical abstract

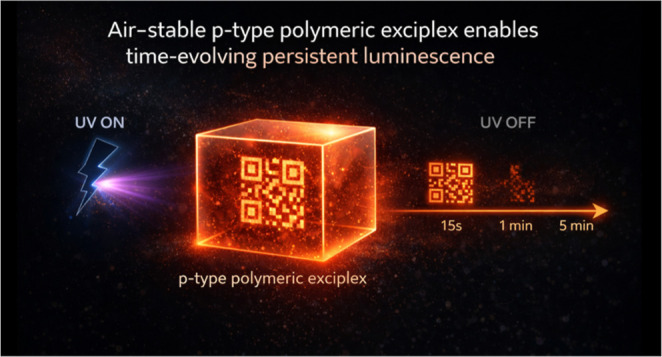

## Introduction

Persistent luminescence (PersL) materials, capable of emitting light long after the removal of an excitation source, have attracted considerable attention in applications such as anticounterfeiting, optical encryption, and information storage.^1–6^ To date, most PersL materials have been based on inorganic systems, which offer long emission durations and excellent photostability.^7–11^ However, their intrinsic brittleness, high processing temperatures, and lack of flexibility significantly hinder their integration with modern soft electronics and wearable technologies.

Organic PersL materials have emerged as a promising alternative due to their structural tunability, mechanical flexibility, and solution processability.^12–16^ Early developments in organic PersL systems primarily relied on phosphorescence via triplet transitions, where the emission lifetime is typically limited to the millisecond-to-second range.^17–24^ More recently, organic exciplex systems have been proposed, wherein long-lived excited states are constructed through charge separation (CS) and charge recombination (CR) processes. An exciplex is a charge-transfer (CT) excited-state complex formed between a donor and an acceptor, which enables charge separation and delayed recombination processes that are beneficial for persistent luminescence. Exciplex systems can be classified as n-type or p-type depending on the dominant carriers; n-type systems are typically sensitive to oxygen due to electron transport, whereas p-type systems based on hole transport can improve air stability. These so-called Exciplex PersL (EPersL) systems are typically formed via the interaction between electron donor and acceptor components, producing spatially separated radical pairs that recombine over extended time scales to yield delayed emission.

The first reported EPersL system was based on an n-type small molecule exciplex system.^25^ However, this system faces two significant challenges: (i) the small-molecule structures are mechanically fragile and lack processability; and (ii) the n-type semiconductor architecture using electrons as carriers is highly sensitive to oxygen quenching during the afterglow process. Although subsequent work has introduced polymer-based n-type and small-molecule p-type exciplex systems, each approach has only addressed one of these challenges individually.^26–28^ Meanwhile, further development of p-type exciplexes represents a major trend, as they address the issue of EPersL oxygen tolerance fundamentally at the level of carrier dynamics. Therefore, the development of polymer-based p-type exciplex systems has become an urgent priority.

In this work, we report the first polymer-based p-type multicomponent exciplex system that can be synthesized via a simple thermal evaporation method. This system exhibits persistent luminescence lasting over 3 h in nitrogen and 7 min in ambient air. By combining poly­(*N*-vinylcarbazole) (PVK) as the hole-transport polymer host, 2,4,6-*tris*(4-methoxyphenyl)­pyrylium tetrafluoroborate (MeOTPP) as the electron acceptor, and *N*,*N*′-*bis*(1-naphthalenyl)-*N*,*N*′-*bis*-phenyl-(1,1′-biphenyl)-4,4′-diamine (α-NPD) as hole-trapping dopant, a donor–acceptor–trap exciplex structure is formed. This exciplex system not only demonstrates exceptional long afterglow and PersL air stability but also exhibits thermoplastic processability and robust mechanical properties, enabling direct patterning via injection molding. Our work estab-lishes a new materials platform for fabricating ambient-stable, device-compatible organic PersL systems with potential applications in anticounterfeiting devices and photonic components.

## Results and Discussion

### Design and Synthesis

The polymer-based p-type exciplex system is the key to our new design. To this end, we physically blend the donor polymer PVK with the acceptor dopant MeOTPP and the trap dopant α-NPD. The selection of MeOTPP and α-NPD is based on their large difference in HOMO energy levels, allowing for a broader range of suitable polymer hosts (Figure 1a). Furthermore, to achieve products with better application prospects, we selected commonly used polymer materials with good processability in the industry as potential hosts for synthesizing the p-type exciplex. Among them, PVK, with advantages such as good chemical stability, excellent thermal stability, strong hole mobility, and good photoconductivity, is widely used as an optoelectronic material in the fabrication of devices such as organic light-emitting diodes (OLEDs) and organic field-effect transistors (OFETs).^29^ So, it is a suitable raw material for synthesizing exciplexes (Figure 1b). Nonetheless, integrating all three components into an exciplex system remains challenging. Conventional exciplex syntheses rely on melt-cooling or drop-casting.^25–28^ However, most polymers lack a well-defined melting point, rendering melt processing impractical, and solvent-casting can suffer from phase separation when solubility mismatches exist. To overcome these obstacles, we developed a one-pot “boil-up” protocol that yields the exciplex simply by solvent evaporation followed by a brief annealing step (Figures 1c,d and S1). Vigorous boiling gradually removes the solvent while freezing the multicomponent mixture in place, thereby guaranteeing uniform dispersion despite differing solubilities. Subsequent annealing further enhances the afterglow performance. This straightforward, purely physical route enables the rapid preparation of multicomponent exciplex materials while preserving the excellent processability of the polymer matrix.

**Figure 1:**
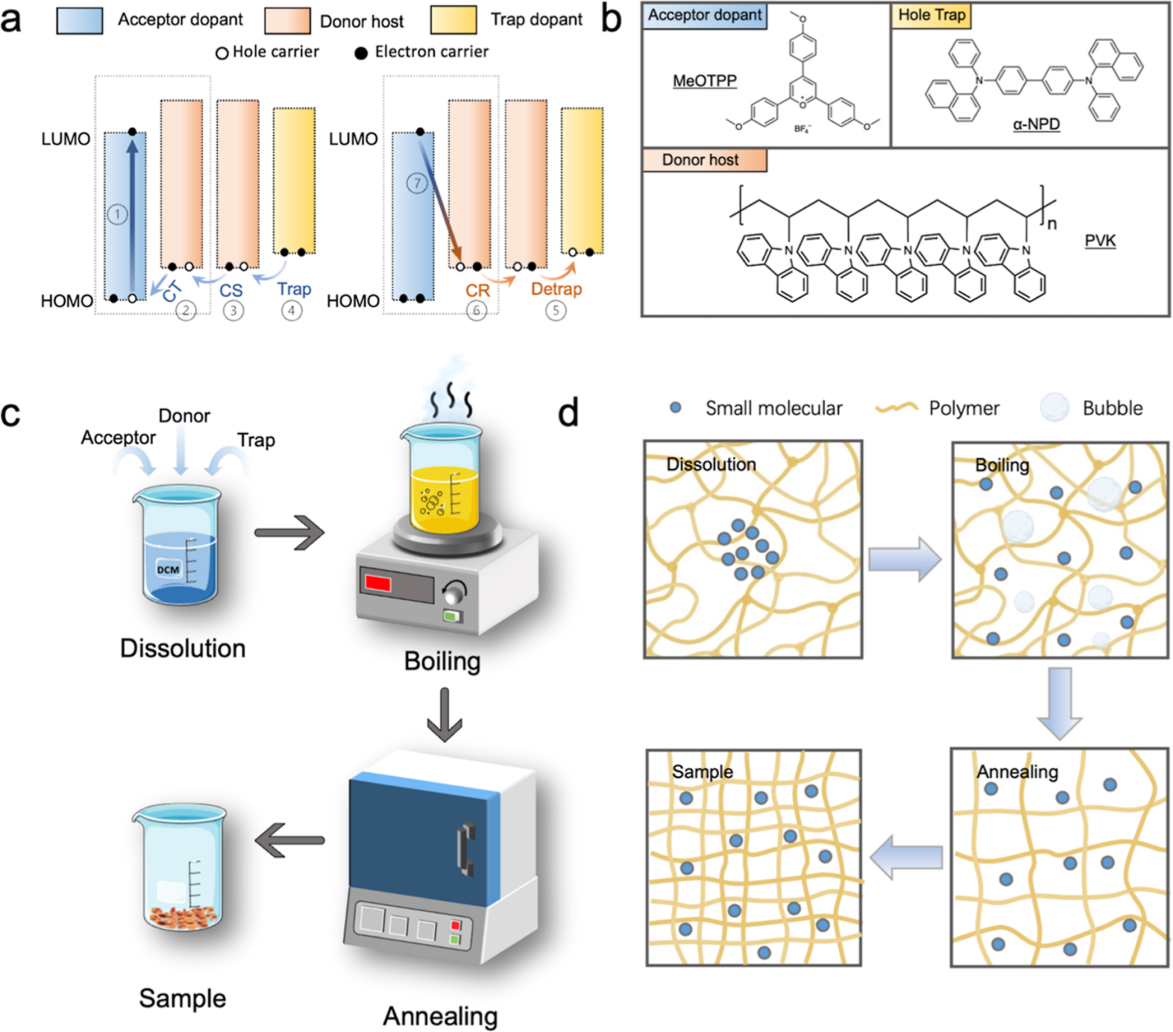
(a) Simplified HOMO–LUMO scheme for electron migration involving excitation, charge transfer (CT), charge separation (CS), trapping, detrapping, charge recombination (CS) and emission process in PVK exciplex; (b) molecular structure of PVK, MeOTPP and α-NPD; (c) scheme of the one-pot “boil-up” protocol used for synthesizing the polymer-based exciplex system; (d) proposed microstructural evolution of the PVK exciplex system during dissolution, boiling, and annealing.

To facilitate further optimization of this newly developed one-pot synthesis method, we conducted a comprehensive investigation into key processing parameters, including heating temperature during solvent evaporation, annealing protocols, and precursor feed ratios. These variables were systematically explored to understand their influence on the PersL and to elucidate the underlying mechanisms.

We first examined the heating temperature during the solvent evaporation stage. As shown in Figure 2a, PersL performance improves with higher synthesis temperature; however, no further enhancement is observed beyond 100 °C compared to 80 °C, indicating that increasing the temperature beyond this range does not lead to additional improvement in molecular dispersion. Fluorescence microscopy images of the PVK exciplex synthesized at 40 °C reveal pronounced molecular aggregation (Figure 2b), attributed to insufficient boiling intensity. This aggregation likely stems from the lower solubility of MeOTPP and α-NPD in dichloromethane (DCM) compared to PVK, making them more prone to phase separation at lower temperatures. Next, we optimized the annealing process. A multistep thermal treatment was introduced to promote PVK crystallization (Figure 2c). Enhanced crystallinity has been reported to increase hole mobility in PVK,^30–32^ which is critical for sustaining charge transport in exciplex systems. As shown in Figure 2d, the relative intensity of the PVK crystallite peak at 2θ = 20.4° varies with annealing conditions. Since further increases in annealing temperature or time did not significantly improve PersL, and in consideration of energy efficiency, a two-stage annealing procedure (200 °C × 1 h + 200 °C × 1 h) can be adopted as being optimal. Finally, we evaluated the impact of feed ratios. At fixed MeOTPP and α-NPD levels (0.01 mmol each), increasing PVK from 0.25 to 2 g effectively extended the hole diffusion pathway and enhanced PersL duration (Figure 2e). However, excessive dilution at higher PVK loadings led to reduced PersL, likely due to incomplete trapping (Figure 2f). A similar optimal condition for α-NPD (0.01 mmol) was also identified (Figure 2g,h). Under these optimized conditions, PVK proved to be a highly effective host for supporting EPersL generation (Figures S2–S5).

**Figure 2:**
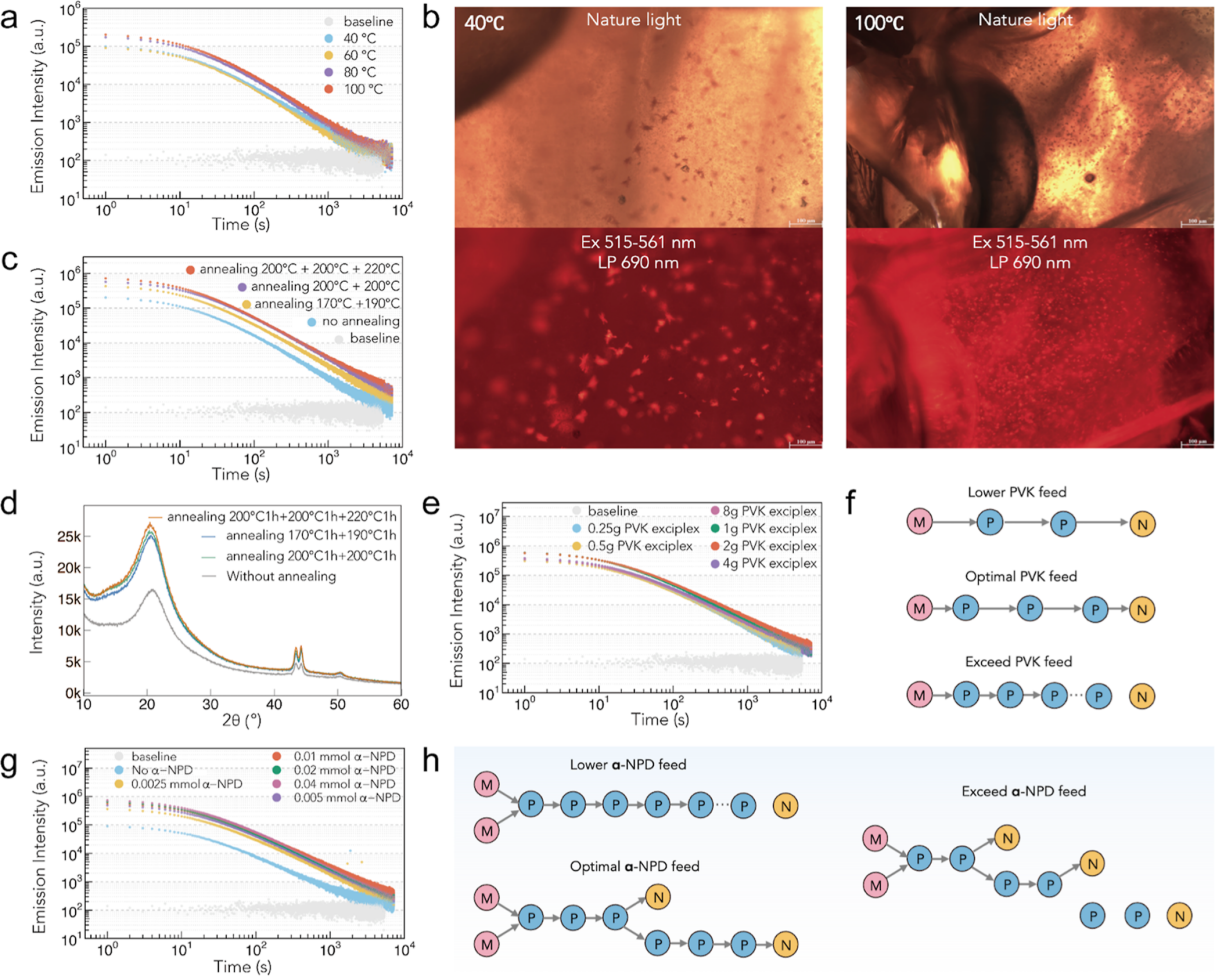
(a) PersL decay curves of PVK exciplex synthesized at different evaporation temperatures; (b) fluorescence microscopy images of the PVK exciplex systems synthesized at 40 and 100 °C under natural light and excitation light source (515–561 nm), with 690 nm long-pass filter; (c) PersL decay curves of a PVK exciplex subjected to various annealing conditions; (d) X-ray diffraction (XRD) patterns of PVK exciplex with/without thermal-annealing treatment; (e) PersL decay curves of PVK exciplex synthesized with varying PVK feed ratio; (f) scheme for carriers diffusion route in the PVK exciplex system for different PVK feed ratios (M = MeOTPP; *P* = PVK; *N* = α-NPD); (g) PersL decay curves of PVK exciplexes synthesized with varying α-NPD feed ratio; (h) scheme for carriers diffusion route in PVK exciplex systems for different α-NPD feed ratios.

### EPersL Properties and Mechanism

The double-logarithmic representation of the PVK exciplex decay curve is shown in Figure 3a. The initial section of the decay follows an exponential decay and is assigned to intrinsic phosphorescence emission. In contrast, the long-time section becomes linear in the double-logarithmic plot, consistent with a power-law behavior associated with carrier diffusion and recombination in exciplex systems. Therefore, to determine the persistent luminescence lifetime of the PVK exciplex system, only the long-time section is used for fitting. Based on the fitting results, the decay curve corresponding equation is *y* = −1.16*x* + 7.03. As shown in Figure S2, the baseline emission intensity, determined from the ambient background signal under the same measurement conditions, remains below 200. Therefore, the PersL duration can be defined as the time when the emission intensity of the sample decreases to 200. By setting *y* = log_10_200, we obtain *x* = log_10_12023, indicating that the PersL duration of the PVK exciplex system is approximately 3 h and 20 min. Additionally, the orange afterglow was visibly recorded for over 1 h using a standard consumer digital camera (Figure 3b). As shown in the absorption spectrum (Figure 3c), the PVK exciplex system exhibits a broad absorption band covering the UV region and extending across nearly the entire visible spectrum. The emission spectrum (Figure 3d) shows a broad emission peak centered at 650 nm, which is distinct from the emission profiles of the individual components. This observation is consistent with the formation of a new exciplex energy level within the PVK exciplex system.

**Figure 3:**
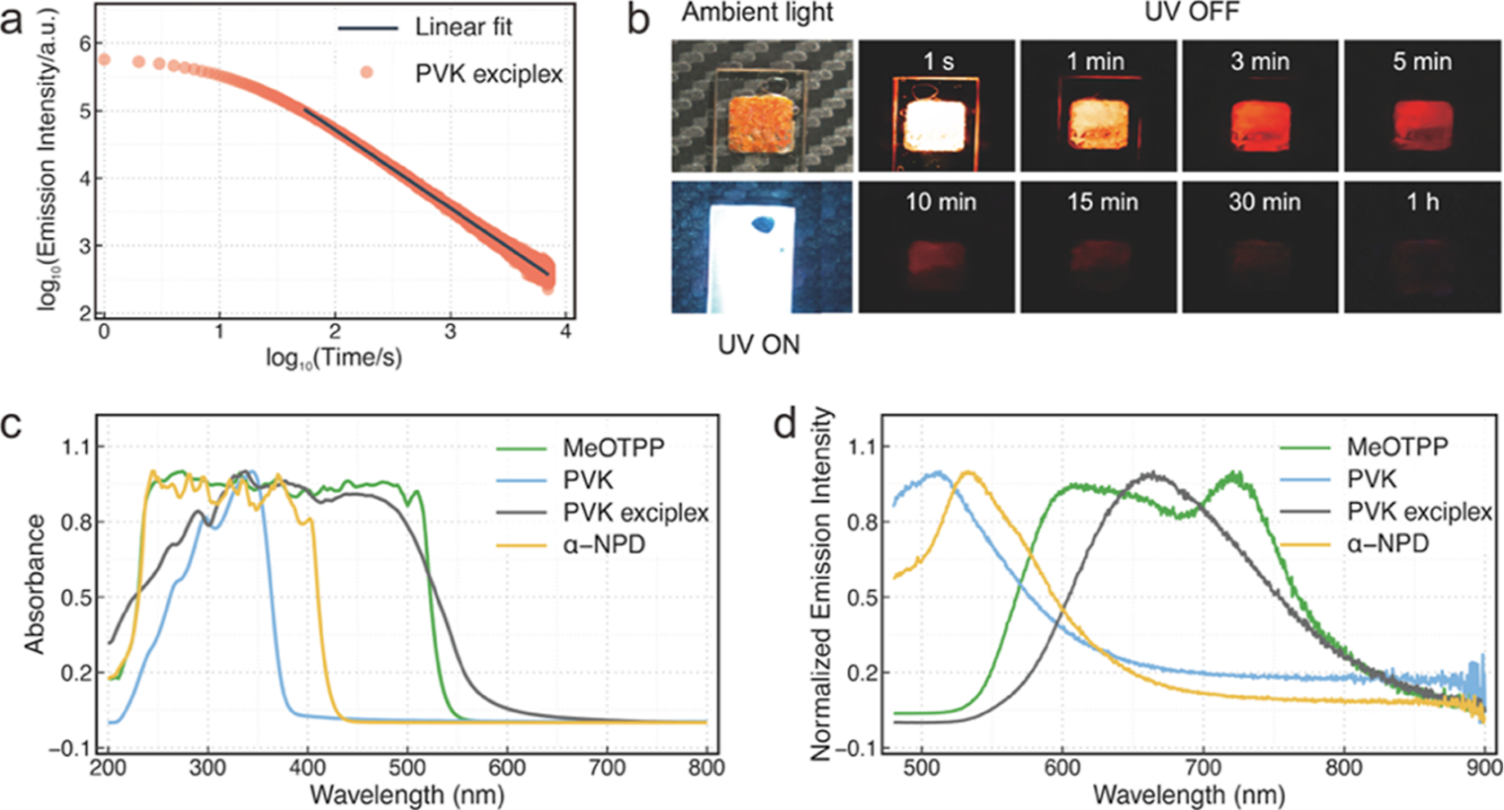
(a) Double-logarithmic representation of the PVK exciplex decay curve with linear fitting of the long-time power-law section; (b) photographs of the PVK exciplex under ambient light, UV excitation, and at various time intervals after turning off the UV light; (c) absorption spectra of PVK exciplex system, PVK, MeOTPP and α-NPD; (d) normalized emission spectra of PVK exciplex system, PVK, MeOTPP and α-NPD (excitation: 468 nm; under inert atmosphere of N_2_).

The HOMO and LUMO energy levels of all components were primarily determined from electrochemical measurements (Figures 4a and S6), while the band gaps derived from absorption spectra (Figures 3c and S7) are used as approximate optical estimates. These values are consistent with computational results (Figure S8) and support the overall energy level alignment. The specific process is illustrated in Figures 4b and S9. Upon photoexcitation, excitons are primarily generated on MeOTPP, followed by charge transfer to PVK (1). The generated holes then diffuse along the PVK chains through a charge separation process (2). The introduction of α-NPD serves as a trap for charge carriers, prolonging the CS process (3). Subsequently, the holes are released from α-NPD (4), continue to diffuse through the PVK matrix, and gradually undergo charge recombination (CR) with MeOTPP (5), ultimately resulting in orange emission (6) with an extended lifetime. This gradual recombination and light emission process produces the exciplex persistent luminescence (EPersL) of the system. As shown by the electrostatic potential (ESP) analysis in Figure 4c, α-NPD and *N*-vinylcarbazole (VK) exhibit high electron density around their benzene rings (highlighted in orange). And MeOTPP shows high positive ESP values at the hydrogen atoms of its methyl groups and at the oxygen atoms of its aromatic rings (highlighted in blue). The strong electrostatic interaction between the donor and acceptor facilitates interfacial charge transfer, which is essential for exciplex formation. The subsequent CS and trapping processes are evidenced by ESR spectra (Figure 4d,e), where a significant increase in radical concentration is observed under light excitation. After the excitation is turned off, the slow intensity decay of the radical indicates the occurrence of detrapping and CR processes within the system.

**Figure 4:**
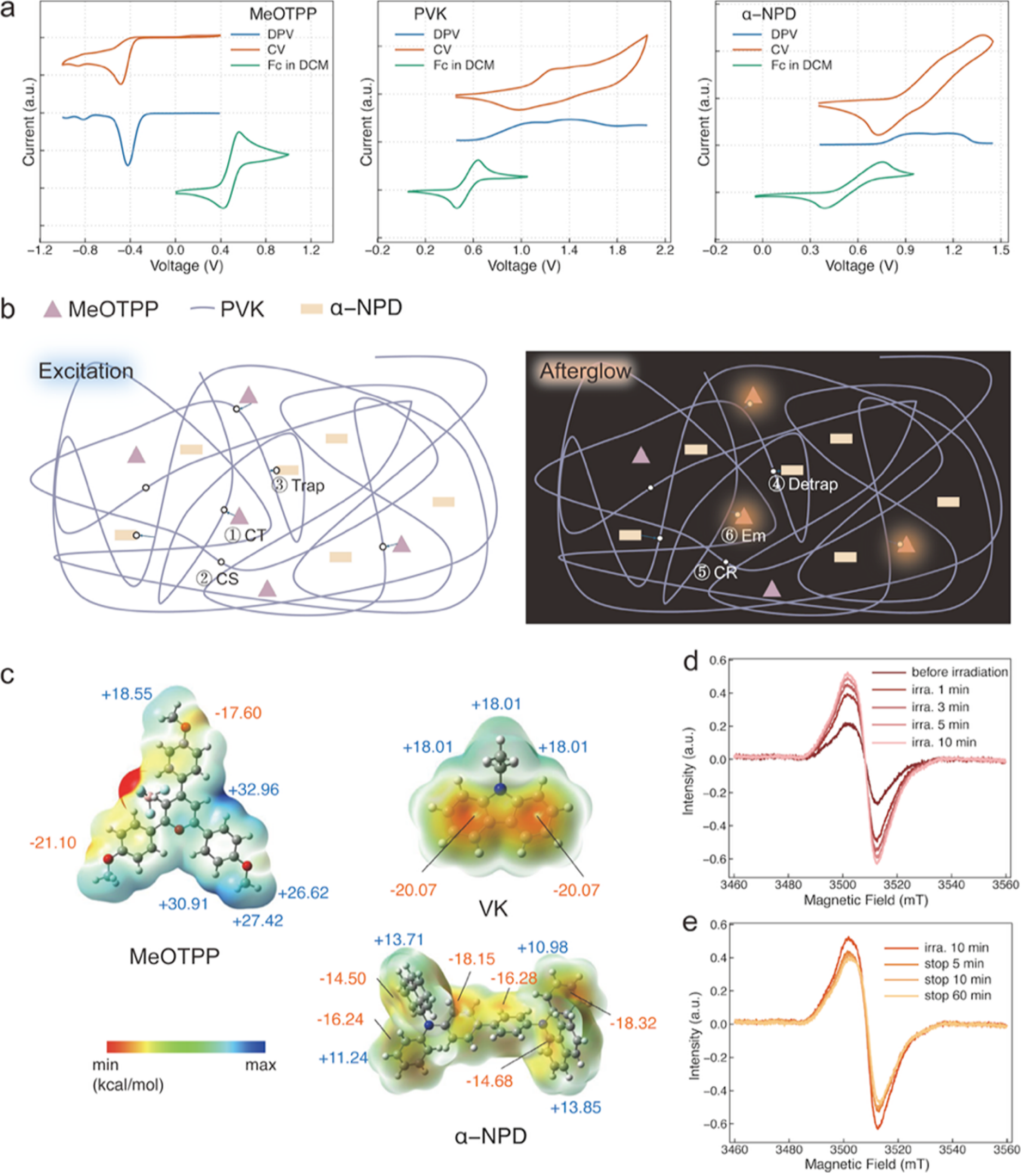
(a) Electrochemical characterization: Cyclic voltammetry (CV) and differential pulse voltammetry (DPV) for MeOTPP, PVK and α-NPD; (b) schematic illustration of the EPersL process in the PVK exciplex system; (c) electrostatic potential (ESP) maps of MeOTPP, α-NPD, and VK; ESR spectra of the PVK exciplex system (d) before and during the excitation, (e) after excitation.

### Carrier Transport and Dynamics

In exciplex systems, a new exciplex energy state (E) is generated during the charge transfer (CT) process, and its position and characteristics have a significant impact on PersL performance.^33,34^ The emission from the E state originates from the electron recombination between the LUMO of the acceptor and the HOMO of the donor (Figure 5a,d). Therefore, to investigate how the E state influences the EPersL, we designed a set of control systems using polymer hosts with different HOMO energy levels—specifically, polyvinyl chloride (PVC) and 4,4’-(1-methylethylidene)­bis­[phenol] (U100)—as comparative examples (Figures S10–S12).

**Figure 5:**
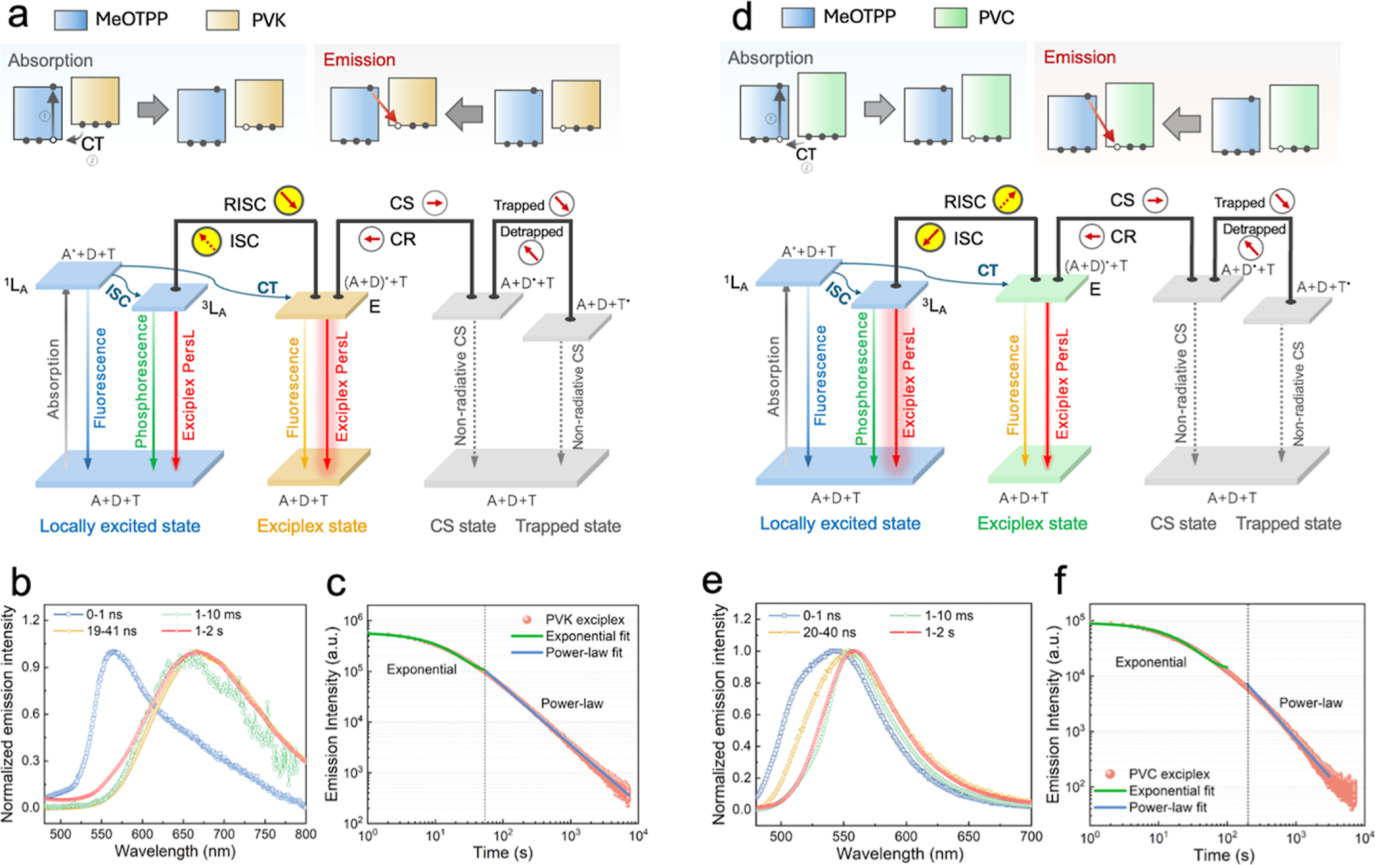
Jablonski diagram and schematic illustration of proposed CT processes occurring between donor and acceptor in PVK (a) and PVC (d) exciplex systems (^1^LA and ^3^LA: singlet and triplet state of locally excited state, respectively; E: exciplex energy state; ISC: intersystem crossing; RISC: reverse ISC; CT: charge transfer; CS: charge separation; CR: charge recombination); Time-resolved emission spectra of PVK (b) and PVC (e) exciplex systems at room temperature; PersL decay curves of PVK (c) and PVC (f) exciplex systems after excitation at 468 nm for 5 min in N_2_ atmosphere.

Time-resolved emission spectra were collected from PVK and PVC exciplex systems over time scales ranging from nanoseconds to seconds, as shown in Figures 5b,e, and S13–S17. Two distinct fluorescence signals were sequentially observed in the nanosecond range, first attributed to the acceptor fluorescence (0–1 ns), followed by exciplex fluorescence (∼20–40 ns). In the millisecond range, a single band indicates the phosphorescence emission from the triplet states of the acceptor (^3^L_A_), while the band observed in the 1–2 s range is attributed to EPersL. As shown by the decay curves, the PersL lifetime of the PVK exciplex is significantly longer than that of the PVC exciplex (Figure 5c,f) and U100 exciplex (Figures S18–S20).

Notably, in the PVK exciplex, the energy of E is lower than ^3^L_A_ (Figure 5a). This configuration ensures that more energy flows toward E, providing sufficient energy to participate in the CS, trap, detrap, and CR processes. As E is at a lower energy, the energy returning to it after the CR process is more likely to undergo radiative transition. Thus, the EPersL peak position overlaps with the fluorescence peak from the exciplex state (Figure 5b). PVC exciplex present a different case, where E is higher than ^3^L_A_ (Figure 5d). This results in a limited energy reserve in E, reducing the energy available for CS and CR processes, thereby leading to shorter PersL performance. Since the E transfers energy to the ^3^L_A_ via intersystem crossing (ISC), much of the energy involved in the CS and CR processes also flows to the ^3^L_A_ state. As shown in Figure 5e, the EPersL peak position nearly coincides with the phosphorescence peak from the locally excited state. Furthermore, PVC exciplexes exhibit more extended exponential decays (Figure 5c,f, and Tables S1–S4). This may be due to the lower energy of ^3^L_A_, which causes more PersL energy to pass through it before emission, thereby exhibiting more characteristics of a phosphorescence decay. Therefore, it can be seen that the emitting state is not necessarily a purely CT state. In certain cases, the contribution of the CT component to the emission can be used to qualitatively evaluate the direction of energy flow and its influence on the persistent luminescence performance.

The differences in PersL performance among the polymer-based exciplex systems originate not only from variations in energy level alignment but also from the intrinsic charge transport properties of the host materials. ESR measurements reveal that the concentration of charge carriers generated in the PVK exciplex is significantly higher than that in the PVC exciplex (Figure S21), highlighting the superior carrier mobility of PVK. Additionally, although the energy gap between the HOMO of the donor and the LUMO of the acceptor in the U100 system is smaller than that of the PVC system (Figure S12), virtually no PersL emission is observed when the polyester-based polymer U100 is employed as the host (Figure S18). This can be attributed to the presence of strongly electron-withdrawing ester groups in the U100 backbone, which compromise its electron-donating ability and hinder the effective formation of exciplex.

In the PVK exciplex, the key energy levels involved in optical activity lie below the redox potential of oxygen (−3.5 eV), as shown in Figure 6a. Specifically, the LUMO level of the acceptor MeOTPP resides at −3.94 eV, effectively preventing electron transfer to O_2_ and thereby enabling ambient-stable PersL. In contrast, n-type exciplex systems often exhibit LUMO levels above −3.5 eV, leading to severe quenching in air.^26^ Furthermore, hole-dominated transport in the p-type system reduces the involvement of electrons that are susceptible to oxygen quenching, further contributing to the improved air stability. The PersL decay profiles of the PVK exciplex, recorded under identical conditions in air and nitrogen (Figure 6b), confirm its impressive PersL oxygen tolerance. Although shortened in air, the PVK exciplex produces PersL emission for over 7 min. Furthermore, the emission and excitation spectra remain nearly unchanged in air compared to nitrogen (Figures 6c and S22), and the material exhibits outstanding photostability under continuous 468 nm irradiation (Figure S23), highlighting its practical potential for ambient applications.

**Figure 6:**
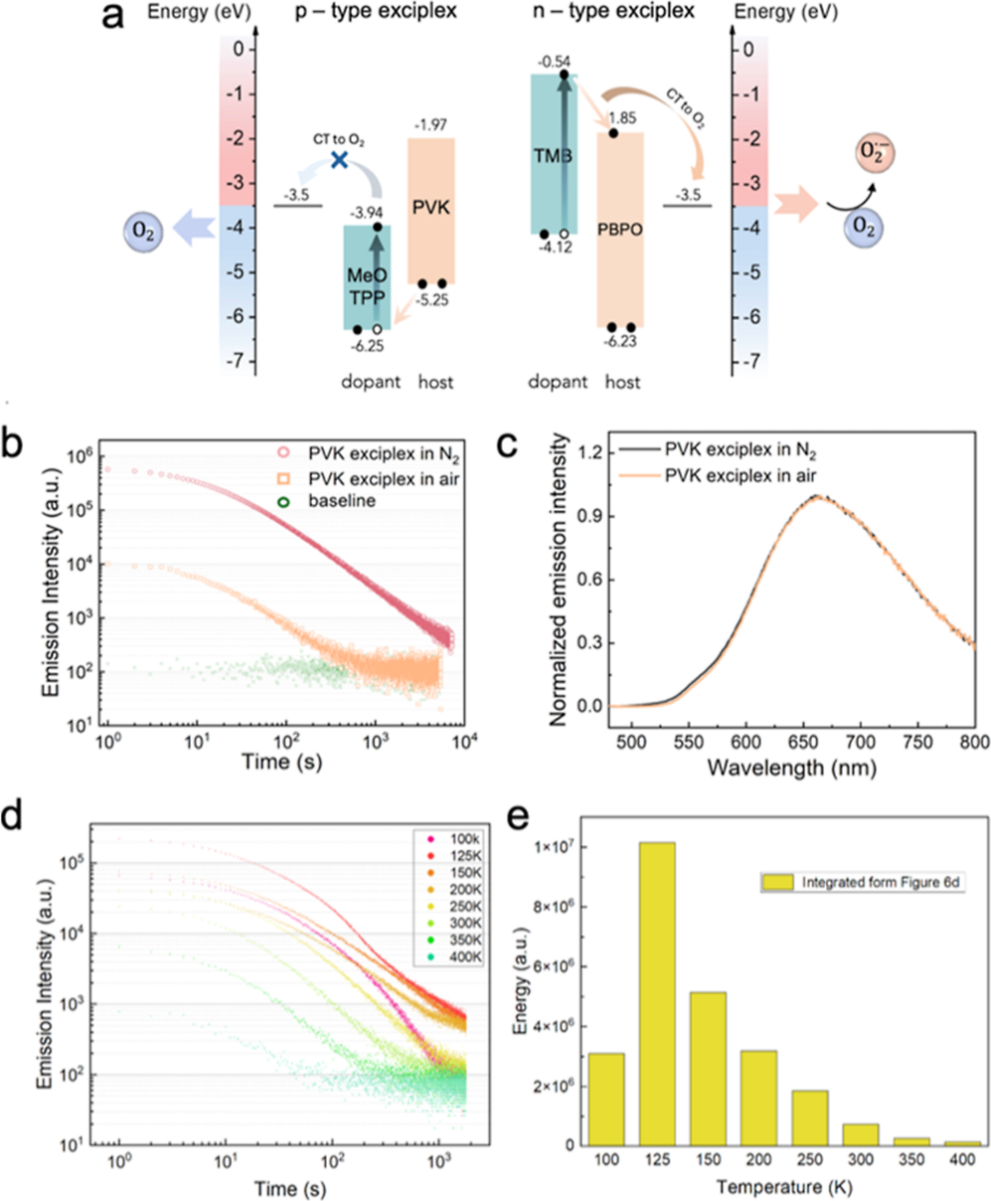
(a) Energy level diagrams of p-type and n-type exciplex systems with respect to the redox potential of molecular oxygen (−3.5 eV); (b) Comparison of PersL decay curves of PVK exciplex after excitation at 468 nm for 5 min in ambient air and N_2_ atmosphere; (c) normalized emission spectra of PVK exciplex measured in ambient air and N_2_ atmosphere; (d) Temperature-dependent PersL decay curves of the PVK exciplex (100–400 K); (e) integrated emission energy derived from the temperature-dependent PersL decay curves.

To further investigate the carrier recombination kinetics underlying the EPersL behavior, temperature-dependent PersL decay measurements were conducted for the PVK exciplex system in the range of 100–400 K (Figure 6d). The results reveal a clear temperature dependence of the PersL lifetime, showing an initial increase followed by a decrease in total PersL duration with rising temperature. From a thermodynamic perspective, the suppression of nonradiative transitions at low temperatures allows more stored energy to be funneled into radiative PersL, which explains the observed lifetime reduction between 200 and 400 K. However, from a kinetic standpoint, lower temperatures hinder the detrapping and recombination rates of carriers, thereby regulating the energy release process. The slope of each decay curve roughly reflects the energy release rate. Notably, a marked change in slope between 200 and 250 K underscores the influence of temperature on the carrier dynamics. At excessively low temperatures (e.g., 100 and 125 K), a portion of trapped carriers remains immobilized in traps, leading to a sharp drop in PersL intensity. Additionally, during the excitation, energy release occurs concurrently with energy storage, thereby influencing the total energy retained postirradiation. To quantify this, the decay curves in Figure 6d were integrated to yield the total released energy at each temperature, as shown in Figure 6e. Apart from the incomplete detrapping at 100 K, the integrated energy values across the remaining temperatures reflect the amount of energy successfully stored during the excitation. A gradual decrease in stored energy with increasing temperature suggests that temperature not only governs the release kinetics of exciplex but also modulates energy capture efficiency during excitation. This dual influence offers a new mechanistic insight into exciplex behavior and highlights the potential of such systems for temperature-sensing applications.

## Processability, Mechanical Properties and Applications

Since PVK acts as the host in the exciplex system, the mechanical properties of the system are primarily dictated by the characteristics of PVK. The PVK polymer is amorphous and isotropic in its glassy state, which gives the exciplex an appreciable level of transparency (Figure S24). Additionally, the processability of PVK enables the PVK exciplex to be easily molded into specific shapes in ambient air using thermoforming techniques (Figure 7a).

**Figure 7:**
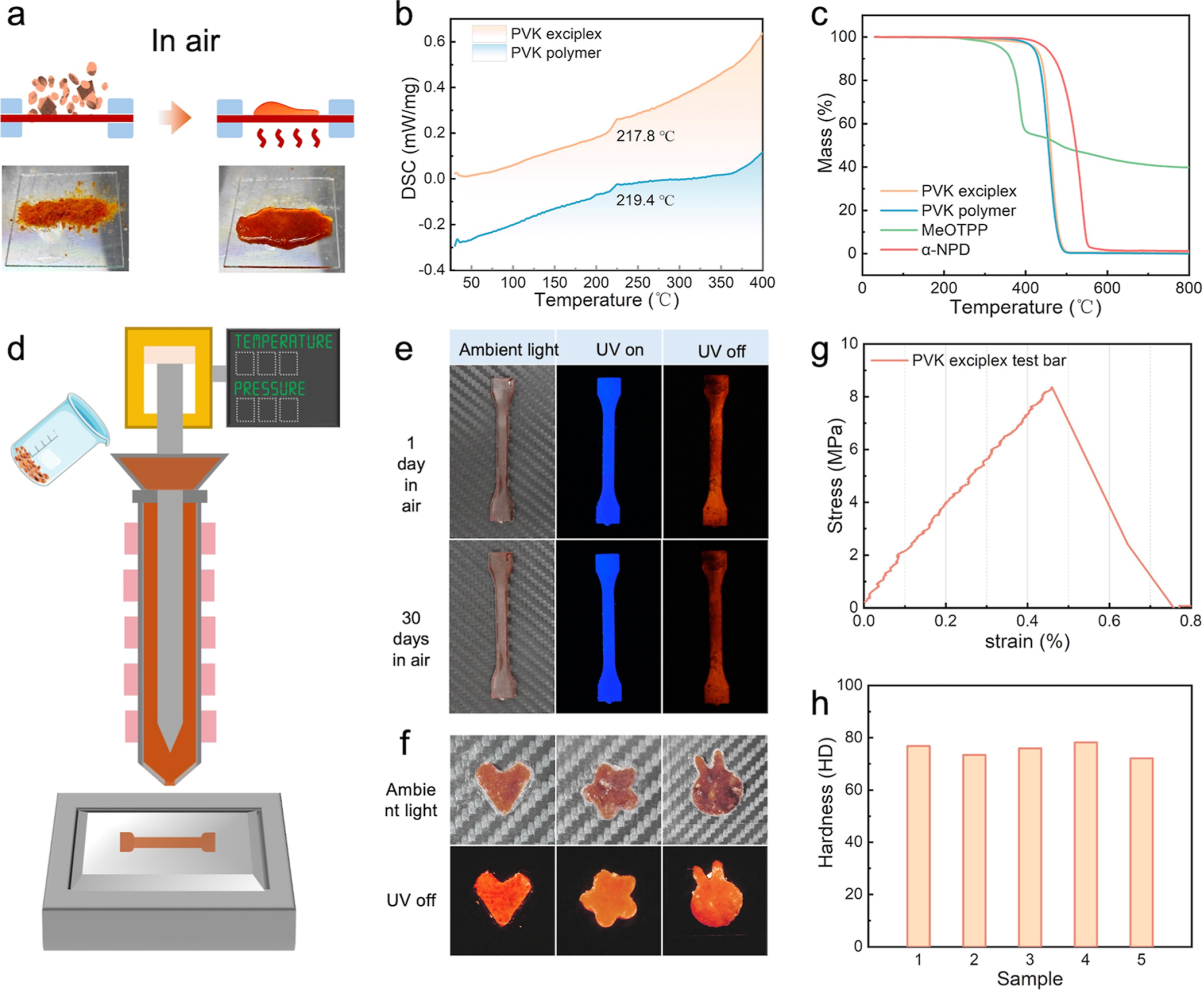
(a) The thermoplasticity of the PVK exciplex during heat treatment in air enables its processability; (b) differential scanning calorimetry (DSC) of PVK polymer and PVK exciplex; (c) thermogravimetric (TG) curves of PVK exciplex, PVK polymer, MeOTPP and α-NPD; (d) scheme of the injection molding technique used to produce the shaped PVK exciplex; (e) comparison of digital images of the PVK exciplex tensile test specimen produced using injection molding techniques, stored in air for 1 day and 30 days, under ambient light and with UV on/off; (f) digital images of the PVK exciplex processed into different shapes, captured under ambient light and with UV off; (g) tensile stress–strain curve of a PVK exciplex tensile test specimen; (h) shore hardness HD test of a PVK exciplex tensile test specimen.

Figure 7b presents the differential scanning calorimetry (DSC) results, which show that the glass transition temperature of the PVK exciplex system is 216.6 °C, closely matching with the PVK polymer (214.1 °C). This suggests that the PVK exciplex system exhibits robust mechanical properties under ambient temperature. Moreover, thermogravimetric (TG) analysis shown in Figure 7c proves the thermal stability of the PVK exciplex system. Among the PVK exciplex system and its components, MeOTPP exhibits the lowest thermal decomposition temperature, yet it remains stable above 300 °C. This allows the PVK exciplex to maintain a molten state without decomposition, making it well-suited for injection molding techniques (Figure 7d), a widely used method in polymer processing. As shown in Figures 7e and S25, tensile test specimens with specific shapes can be produced under controlled temperature conditions. After storage in ambient conditions for 30 days, the material still exhibits PersL when excited by UV light. This proves that the PVK exciplex product is resistant to phase separation under ambient conditions, therefore demonstrating excellent long-term stability. Furthermore, simple die-cutting, signet and quartz groove (Figures 7f and S26–S28) can be used to create specific shapes, highlighting its exceptional processability. The mechanical properties of the molded tensile test specimens were evaluated (Figure S29). The tensile stress–strain curve (Figure 7g) indicates that the tensile test specimen is rigid, exhibiting high resistance to elastic deformation. The calculated Young’s modulus of 1.96 GPa suggests that the mechanical properties of the PVK exciplex are comparable to those of Bakelite or aluminum–nickel alloys (Table S5). It also shows that the tensile test specimen exhibits a maximum tensile stress of 8.36 MPa and a tensile strain of 0.46% at fracture. Additionally, the Shore hardness (HD) test (Figure 7h) shows that the PVK exciplex product has a hardness of 75.3, comparable to that of a hard hat (Table S6). This hardness allows a 30*30*1 mm thin plate to easily support a 50 g weight (Figure S30). These results collectively demonstrate that the PVK exciplex system not only exhibits desirable optical and thermal properties but also fulfills practical mechanical requirements for real-world processing and device integration.

The distinct difference in afterglow lifetime of the PVK exciplex in air versus in an inert atmosphere provides an excellent feature for anticounterfeiting applications. To validate the potential of the constructed PVK exciplex PersL system in information security, three PMMA plates (9 mm * 9 mm * 4 mm) were engraved and used as substrates for fabricating a multilayer QR-code anticounterfeiting device (Figure 8a). Different luminescent materials were then filled into the pre-engraved grooves: the bottom layer was the CaAlSiN_3_:Eu^2+^ phosphor, which does not exhibit PersL but shows an emission peak similar to that of the PVK exciplex under UV excitation; the middle layer consisted of PVK exciplex encapsulated in an inert atmosphere; and the top layer was PVK exciplex exposed to air (Figure 8b). Under ambient light, the device appeared uniformly orange of all three layers, rendering the QR code unreadable. Upon UV excitation, all three layers emitted simultaneously, and the information remained hidden. Nevertheless, the multilayer design enabled the device to display time-evolving QR-code patterns after the excitation was stopped. Immediately after turning off the excitation, only the middle and top PVK exciplex layers glowed, forming a complete, readable QR code. Although emission from the air-sensitive top layer rapidly decayed, the QR code could still be read within 15 s due to comparable intensities from the top and middle layers. Beyond 15 s, the top layer emission diminished significantly, and although faint luminescence was still visible, the QR information became unreadable. Eventually, only the long-lived afterglow of the middle layer remained, before disappearing completely (Figure 8c). Throughout this process, the QR code information could only be effectively read within a narrow time window of ∼15 s after excitation. This temporal dependence imparts a deceptive effect, creating the illusion that the device carries no information, thus offering strong protection against unauthorized access without prior knowledge of the mechanism (Figure 8d). This design realizes time-dependent dynamic encryption of information and demonstrates the unique potential of p-type polymer exciplex PersL materials for advanced anticounterfeiting applications.

**Figure 8:**
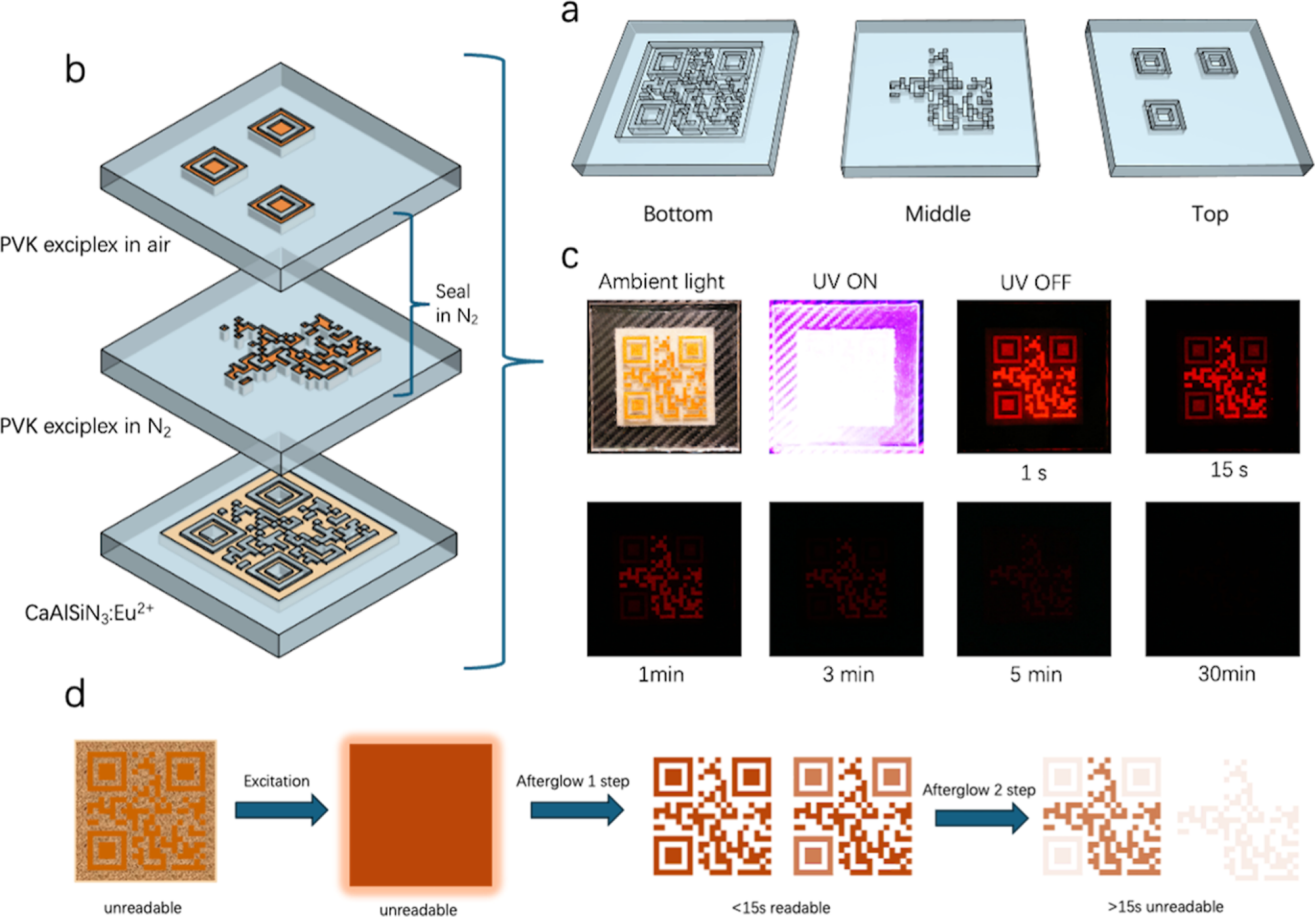
(a) Engraved PMMA plates. (b) Schematic illustration of the assembly of the multilayer QR-code anticounterfeiting device. (c) photographs of the multilayer QR-code device under ambient light, UV excitation, and afterglow at different time. (d) schematic diagram of the anticounterfeiting mechanism.

## Conclusion

In this work, a polymer-based p-type multicomponent exciplex system was constructed for the first time, achieving visible-range PersL lasting over 3 h in nitrogen and more than 7 min in air. By employing PVK as the donor host polymer, MeOTPP as the acceptor dopant, and α-NPD as the trapping dopant, a ternary system with favorable energy level alignment and carrier modulation was developed. A facile one-pot boiling–annealing strategy was introduced, enabling the scalable fabrication of exciplex materials with excellent processability, thermal stability, and PersL air stability, compatible with standard polymer processing techniques. Importantly, the mechanistic origin of PersL in this p-type exciplex system was systematically elucidated through a combination of electrochemical analysis, time-resolved emission spectroscopy, temperature-dependent decay kinetics, ESR spectroscopy, and DFT calculations. A multistep excited-state pathway involving charge transfer (CT), charge separation (CS), trap/detrap, and charge recombination (CR) processes was identified. It was found that the relative energy positioning between the exciplex state (E) and the triplet state (^3^LA) governs the energy flow and recombination probability, while the carrier mobility of the polymer host modulates the lifetime and efficiency of PersL. This dual control mechanism represents a new structure–function paradigm for exciplex systems. Furthermore, temperature-dependent PersL studies further reveal that both energy storage and release processes are thermally tunable, offering mechanistic insights into carrier dynamics and opening avenues for temperature-sensing applications. The resulting PVK exciplex exhibits not only long-lived emission and robust photostability, but also superior mechanical performance and moldability. It can be readily shaped into complex geometries via thermoforming and injection molding, maintaining EPersL properties even after prolonged exposure to ambient conditions. The difference in afterglow lifetimes of the PVK exciplex in air and inert atmospheres can also be ingeniously exploited for designing anticounterfeiting applications. This study not only establishes a new class of polymer-based p-type exciplex, but also offers an experimentally validated mechanistic framework to guide the future design of functional long-persistent organic luminescence materials.

## Experimental Procedures

### Characterization

Cyclic voltammetry (CV) measurements were performed on a CHI660E voltametric analyzer in dried and oxygen-free DCM (PVK, PVC, U100) or DMF (TPP), using 0.1 M tetrabutylammonium hexafluorophosphate (TBAPF_6_) as a supporting electrolyte. A platinum fiber was used as working electrode, glassy carbon as counter electrode, and Ag/Ag^+^ as reference electrode. The cyclic voltammetry curves were recorded at a scan rate of 50 mV/s, and the differential pulse voltammetry (DPV) curves were obtained with a pulse width (Δ*E*_pulse_) of 0.05 V. Absorption spectra were collected with a Techcomp UV2600 spectrophotometer. Decay profile and steady-state photoluminescence were recorded on an Edinburgh Instrument FLS1000 spectrometer equipped with a 450 W xenon lamp as an excitation source. Time-resolved emission spectra were measured with the same spectrofluorometer equipped with a 450 nm EPL pulsed laser as excitation sources (0–50 ns, 0–1 μs, 0–10 μs, 0–100 μs, 0–1 ms) and a 60 W μs flash lamp (0–10 ms), and measured with Ocean Insight QEPro fiber optic spectrometer (1–2 s). Photographs of samples were recorded using a commercial Sony α6700 camera. Electron spin resonance (ESR) measurements were carried out on a Bruker EMXplus-6/1 spectrometer before and after 468 nm excitation. Thermogravimetric (TG) analyses were performed on a Netzsch STA 449F3 instrument in flowing N_2_ with a heating rate of 10 °C/min. Differential scanning calorimetry (DSC) analyses were measured by a NETZSCH DSC214 Polyma instrument. Fluorescence micrographs were performed on a Leica Microsystem DM6B upright microscope. X-ray diffraction (XRD) data were collected on a D8 ADVANCE powder diffractometer (Bruker, Germany) with Cu Kα radiation (λ = 1.5405 Å) at room temperature in the range of 10°<2θ < 80° Tensile tests were made with an INSTRON 5982 tensile testing machine (INN Demokritos, Greece). Hardness data were collected by a Haibao HS-D Shore hardness tester.

### Chemicals and Materials

2,4,6-*Tris*(4-methoxyphenyl)­pyrylium tetrafluoroborate (MeOTPP) was purchased from Sigma-Aldrich and purified by recrystallization. Poly­(*N*-vinylcarbazole) (PVK) was purchased from HuaXia Chemical Reagent Co. Ltd. (Chengdu) and purified by reprecipitation. *N*,*N*′-*Bis*(1-naphthalenyl)-*N*,*N*′-*bis*-phenyl-(1,1′-biphenyl)-4,4′-diamine (α-NPD) was purchased from Sigma-Aldrich and purified by recrystallization. Dichloromethane (DCM) was purchased from Shanghai Macklin Biochemicals Co. Ltd. and purified by redistillation. Polyvinyl chloride (PVC) and 1,3-benzenedicarboxylic acid, polymer with 1,4-benzenedicarboxylic acid and 4,4’-(1-methylethylidene)*bis*[phenol] (U100) were purchased from Langfang Liangying Plasticization and purified by reprecipitation. CaAlSiN_3_:Eu^2+^ was purchased from Shenzhen Looking Long Technology Co., Ltd.

### Theoretical Calculations

Density functional theory (DFT) calculations were conducted utilizing the Gaussian 16W program.^35^ Geometry optimizations of MeOTPP, α-NPD, VK and PVK were executed at the B3LYP-D3­(BJ) level of theory with 6–31G*basis sets.^36^ Subsequently, the electrostatic potential (ESP) was derived. The calculations were conducted using Multiwfn (version 3.8)^37^ and rendered by VMD software (version 1.9.3). A contour surface threshold of 0.001 au was employed to represent the molecular surface, and the local extrema of the electrostatic potential were determined using Multiwfn. Natural orbitals for the ground state, calculated at the B3LYP-D3­(BJ)/6–31G* level of theory, were visualized using VMD with a vcube script.

### Preparation of Polymer Exciplex

A total of 20 mL of dichloromethane, 0.25–8 g of poly­(*N*-vinylcarbazole), 4.68 mg (0.01 mmol) of 2,4,6-*tris*(4-methoxyphenyl)­pyrylium tetrafluoroborate, and 0–23.56 mg (0–0.04 mmol) of *N*,*N*′-*bis*(1-naphthalenyl)-*N*,*N*′-*bis*-phenyl-(1,1′-biphenyl)-4,4′-diamine were combined in a 50 mL beaker. The beaker was subsequently heated to a temperature range of 40–100 °C on a heating plate. Following the complete evaporation of DCM, a multistage annealing treatment was applied, consisting in two heating steps: 1 h treatments at 200 °C, then leave the sample cool at r.t. and 1 h treatments at 200 °C then cooling to r.t. again (Other annealing treatment see Figure S4). The preparation of the PVC and U100 exciplex systems adhered to the same procedure, substituting PVK with PVC or U100, respectively.

### Preparation of PVK Exciplex Products

A small injection molding machine with aluminum molds was used to produce tensile test specimens at 240 °C injection temperature and 0.5 MPa pressure. The total length of the tensile test specimen was 75 mm and the dimensions of other different parts were shown in Figure S36. In addition, 30*30*1 mm thin plates were made in the same way.

Die cuts were used to make heart-shaped, flower-shaped, and rabbit-head-shaped PVK exciplex samples. The PVK exciplex powder was placed on a glass plate lying on a table that was heated to 240–250 °C; then the boundaries of the shapes were die cut to obtain the final products.

## Supplementary Material



## Notes

Additional photographs and optimization studies for PVK exciplex synthesis (evaporation temperature, annealing protocol, and feed ratios); electrochemical (CV/DPV) and optical (absorption) data with detailed HOMO/LUMO calculations; DFT-derived natural orbitals and energy-level diagrams; time-resolved emission spectra (PVK/PVC/U100) and ESR spectra; excitation/emission spectra in N_2_ and air and photostability tests; processing and mechanical characterization (transparent film, sample dimensions, die-cut/stamping, tensile setup, Young’s modulus and Shore hardness) including fitting parameters and supplementary tables (PDF)

## Abbreviations

PersL: persistent luminescence
EPersL: exciplex persistent luminescence
PVK: poly­(*N*-vinylcarbazole)
MeOTPP: 2,4,6-*tris*(4-methoxyphenyl)­pyrylium tetrafluoroborate
α-NPD: *N*,*N*′-*bis*(1-naphthalenyl)-*N*,*N*′-bisphenyl-(1,1′-biphenyl)-4,4′-diamine
CT: charge transfer
CS: charge separation
CR: charge recombination
ESR: electron spin resonance
CV: cyclic voltammetry
DPV: differential pulse voltammetry
ESP: electrostatic potential
DFT: density functional theory
DSC: differential scanning calorimetry
TG: thermogravimetric analysis
XRD: X-ray diffraction
ISC: intersystem crossing
RISC: reverse intersystem crossing
OLED: organic light-emitting diode
OFET: organic field-effect transistor.
