# Supplementary material for: Polymer-Based p‑Type Exciplex Systems for Air-Stable and Processable Organic Persistent Luminescence
Source: JACS Au. 2026 May 26;6(7):3804–15. doi: 10.1021/jacsau.6c00384 (PMC13417281; doi:10.1021/jacsau.6c00384)
Supplement: Supplementary file 1 [file au6c00384_si_001.pdf]

Supporting Information

Polymer-Based p-Type Exciplex Systems for Air-Stable and Processable Organic Persistent Luminescence

Feilong Liu, Liyi Li, Angelo Homayoun All,* Jonathan WC Wong,* and Jean-Claude G. Bünzli,**

Dr. Feilong Liu
Research Center for Eco-environmental Engineering, Dongguan University of Technology, Dongguan 523808, China
Department of Applied Biology and Chemical Technology, The Hong Kong Polytechnic University, Hong Kong SAR 999077, China
Email: feiloliu@polyu.edu.hk

Dr. Liyi Li
Research Center for Eco-environmental Engineering, Dongguan University of Technology, Dongguan 523808, China
Email: liliyi@dgut.edu.cn

Prof. Dr. Angelo Homayoun All
Department of Chemistry, Hong Kong Baptist University, Hong Kong SAR 999077, China
Email: angelo@hkbu.edu.hk

Prof. Dr. Jonathan WC Wong
Research Center for Eco-environmental Engineering, Dongguan University of Technology, Dongguan 523808, China
Email: jwcwong@dgut.edu.cn

Prof. Dr. Jean-Claude G. Bünzli
Department of Applied Biology and Chemical Technology, The Hong Kong Polytechnic University, Hong Kong SAR 999077, China
Email: jean-claude.buenzli@polyu.edu.hk
Institute of Chemical Sciences & Engineering, Swiss Federal Institute of Technology, Lausanne 1015, Switzerland
Email: jean-claude.bunzli@epfl.ch

a) b)

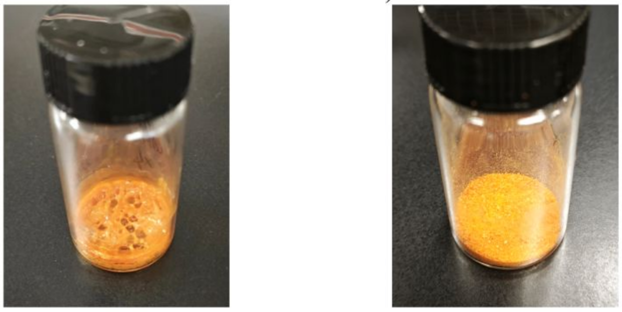

Figure S1. Photograph of synthesized PVK exciplex sample. a) Sample before processing. b) Sample processed into crushed form.

Effect of evaporate temperature on afterglow time

Synthesis temperature	Afterglow time
40 ℃	41 min
60 ℃	38 min
80 ℃	59 min
100 ℃	1 h 5 min

Feed ratio: PVK:MeOTPP:α-NPD=2 g:0.01 mol:0.01 mol.

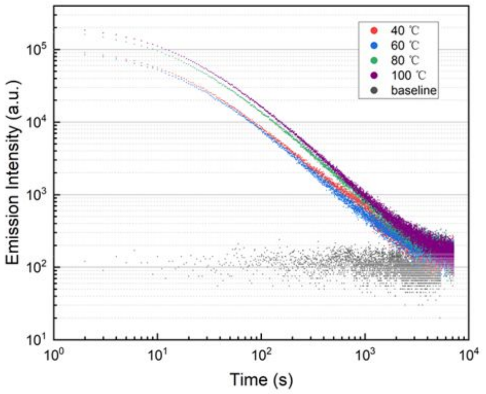

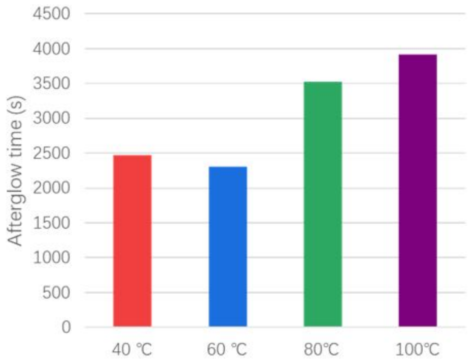

Model	ExpDec1			
Equation	y = A1*exp(-x/t1) + y0			
Plot	40 ℃	60 ℃	80 ℃	100 ℃
y0	20386.02619 ± 642.18691	19379.92705 ± 612.5181	35127.50599 ± 1208.21013	40605.9019 ± 1248.98519
A1	80780.69425 ± 578.25763	75740.01833 ± 575.56894	145169.23753 ± 1224.12398	166102.97448 ± 1195.36891
t1	12.2948 ± 0.31095	11.98284 ± 0.31566	11.49767 ± 0.32395	11.85799 ± 0.29327
Reduced Chi-Sqr	518007.45592	513301.79271	2287103.16588	2209356.13816
R-Square (COD)	0.99857	0.99839	0.99804	0.99856
Adj. R-Square	0.99847	0.99827	0.9979	0.99845

Equation	y = a + b*x			
Plot	40 ℃	60 ℃	80 ℃	100 ℃
Weight	No Weighting			
Intercept	6.14644 ± 0.01229	6.19871 ± 0.01025	6.58227 ± 0.00842	6.7263 ± 0.00762
Slope	-1.11212 ± 0.00444	-1.15927 ± 0.0037	-1.21627 ± 0.00304	-1.24747 ± 0.00275
Residual Sum of Squares	2.52523	1.75571	1.18513	0.97134
Pearson's r	-0.9905	-0.99389	-0.99624	-0.99707
R-Square (COD)	0.98108	0.98781	0.99249	0.99414
Adj. R-Square	0.98107	0.9878	0.99249	0.99414

Figure S2. Optimization of PersL time by varying the heating temperature (40-100 °C) during the evaporation process for PVK exciplex system. The two tables at the bottom provide the fitted initial exponential and subsequent power law. (Excitation: 468 nm, 150 mw, 5 min; Monitored emission wavelength: 650 nm; Atmosphere environment: N_2)

Effect of annealing process on afterglow time

Annealing process	Afterglow time
None	1h 5 min
170℃ 1h + 190℃ 1h	2 h 16 min
200℃ 1h + 200℃ 1h	3 h 20 min
200℃ 1h + 200℃ 1h +220℃ 1h	3 h 25 min

Feed ratio: PVK:MeOTPP:α-NPD=2 g:0.01 mol:0.01 mol. Synthesis temperature: 100 ℃.

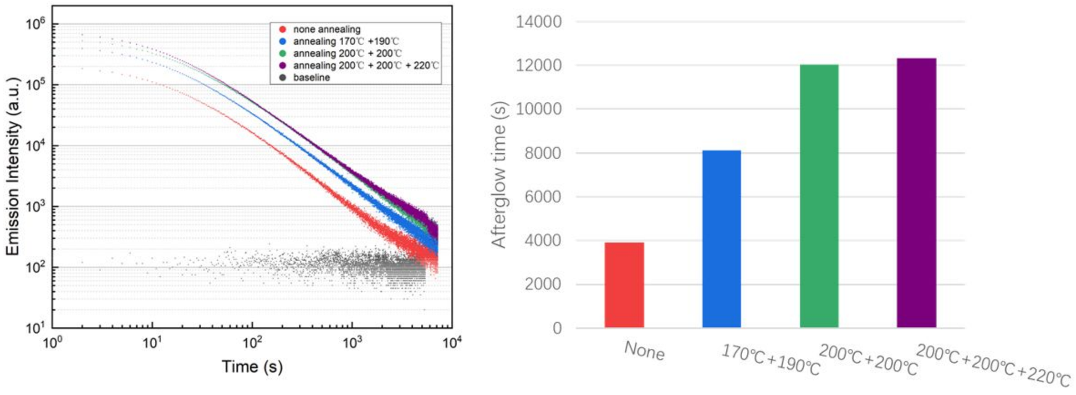

Model	ExpDec1			
Equation	y = A1*exp(-x/t1) + y0			
Plot	none annealing	annealing 170℃ +190℃	annealing 200℃ + 200℃	annealing 200℃ + 200℃ + 220℃
y0	39458.36976 ± 1150.5577	82405.62316 ± 2040.23045	118828.09754 ± 2858.60864	137501.07333 ± 3567.08825
A1	166604.37922 ± 1182.34732	361019.86187 ± 2200.8021	472313.99999 ± 2676.58101	610649.57434 ± 4173.31858
t1	12.0789 ± 0.2836	11.78111 ± 0.23174	12.72364 ± 0.24949	11.32205 ± 0.23908
Reduced Chi-Sqr	2305849.4837	7855667.96326	1.20093E7	2.72268E7
R-Square (COD)	0.99851	0.99892	0.99904	0.99868
Adj. R-Square	0.99841	0.99885	0.99897	0.99859

Equation	y = a + b*x			
Plot	none annealing	annealing 170℃ +190℃	annealing 200℃ + 200℃	annealing 200℃ + 200℃ + 220℃
Weight	No Weighting			
Intercept	6.7238 ± 0.00779	6.96128 ± 0.00512	7.03363 ± 0.00418	7.05011 ± 0.00365
Slope	-1.24653 ± 0.00267	-1.2083 ± 0.00175	-1.15994 ± 0.00121	-1.15775 ± 0.00125
Residual Sum of Squares	2.43946	1.05915	11.11629	0.53961
Pearson's r	-0.99586	-0.99808	-0.9962	-0.99894
R-Square (COD)	0.99173	0.99617	0.99242	0.99787
Adj. R-Square	0.99172	0.99617	0.99242	0.99787

Figure S3. Optimization of PersL time by varying the thermal-annealing treatment in the annealing procedures for PVK exciplex system. (between the heat treatments we leave the sample cool at r.t.). The two tables at the bottom provide the fitted initial exponential and subsequent power law. (Excitation: 468 nm, 150 mw, 5 min; Monitored emission wavelength: 650 nm; Atmosphere environment: N_2)

Effect of PVK feed ratio on afterglow time

PVK	MeOTPP	α-NPD	Afterglow time
0.25 g	0.01 mmol (4.86 mg)	0.01 mmol (5.89 mg)	1 h 31 min
0.5 g	0.01 mmol (4.86 mg)	0.01 mmol (5.89 mg)	1 h 44 min
1 g	0.01 mmol (4.86 mg)	0.01 mmol (5.89 mg)	2 h 34 min
2 g	0.01 mmol (4.86 mg)	0.01 mmol (5.89 mg)	3 h 20 min
4 g	0.01 mmol (4.86 mg)	0.01 mmol (5.89 mg)	2 h 24 min
8 g	0.01 mmol (4.86 mg)	0.01 mmol (5.89 mg)	2 h 27 min

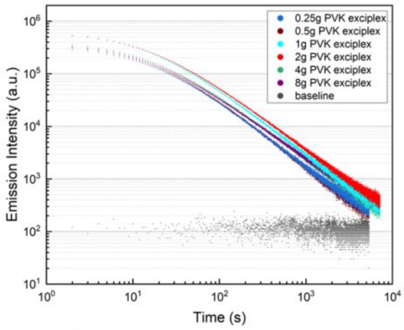

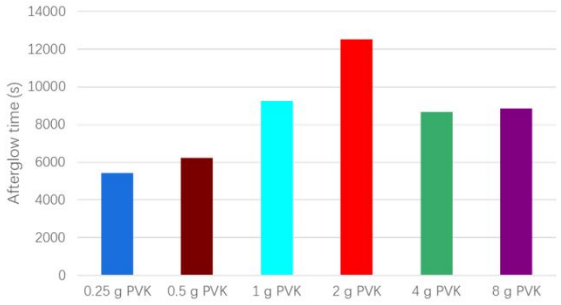

Model	ExpDec1					
Equation	y = A1*exp(-x/t1) + y0					
Plot	0.25 g PVK exciplex	0.5 g PVK exciplex	1 g PVK exciplex	2 g PVK exciplex	4 g PVK exciplex	8 g PVK exciplex
y0	60046.88217 ± 1352.34212	56350.23631 ± 1338.09685	99603.19524 ± 2292.0629	118828.09754 ± 2858.60864	69224.50869 ± 1717.0984	65949.50746 ± 1434.97214
A1	283985.87898 ± 1656.90978	260885.17295 ± 1584.48392	500556.73819 ± 3063.53935	472313.99999 ± 2676.58101	320444.87576 ± 2071.04394	296890.14967 ± 1759.64126
t1	13.74887 ± 0.24023	13.98605 ± 0.25902	13.17603 ± 0.23047	12.72364 ± 0.24949	13.85704 ± 0.27045	13.74307 ± 0.24382
Reduced Chi-Sqr	5768273.39039	5359278.33183	1.88327E7	1.20093E7	9079683.21449	6503057.71644
R-Square (COD)	0.99875	0.99863	0.99867	0.99904	0.99845	0.99871
Adj. R-Square	0.99868	0.99856	0.99861	0.99897	0.99838	0.99864

Equation	y = a + b*x					
Plot	0.25 g PVK exciplex	0.5 g PVK exciplex	1 g PVK exciplex	2 g PVK exciplex	4 g PVK exciplex	8 g PVK exciplex
Weight	No Weighting					
Intercept	7.0335 ± 0.00635	6.9352 ± 0.00619	7.16493 ± 0.00478	7.03363 ± 0.00418	6.9378 ± 0.00494	6.80233 ± 0.0048
Slope	-1.27491 ± 0.00218	-1.23583 ± 0.00212	-1.23601 ± 0.0016	-1.15994 ± 0.00121	-1.18679 ± 0.0017	-1.13683 ± 0.00165
Residual Sum of Squares	1.33408	1.26845	0.75626	11.11629	0.80883	0.76183
Pearson's r	-0.99748	-0.99745	-0.99848	-0.9962	-0.99823	-0.99819
R-Square (COD)	0.99497	0.99491	0.99696	0.99242	0.99647	0.99638
Adj. R-Square	0.99496	0.9949	0.99696	0.99242	0.99647	0.99638

Figure S4. Optimization of PersL time by varying the PVK feed ratio in PVK exciplex system. The two tables at the bottom provide the fitted initial exponential and subsequent power law. (Excitation: 468 nm, 150 mw, 5 min; Monitored emission wavelength: 650 nm; Atmosphere environment: N_2)

Effect of α-NPD feed ratio on afterglow time

PVK	MeOTPP	α-NPD	Afterglow time
2 g	0.01 mmol (4.86 mg)	0 mmol	44 min
2 g	0.01 mmol (4.86 mg)	0.0025 mmol	1 h 59 min
2 g	0.01 mmol (4.86 mg)	0.005 mmol	2 h 1 min
2 g	0.01 mmol (4.86 mg)	0.01 mmol	3 h 20 min
2 g	0.01 mmol (4.86 mg)	0.02 mmol	2 h 43 min
2 g	0.01 mmol (4.86 mg)	0.04 mmol	2 h 24 min

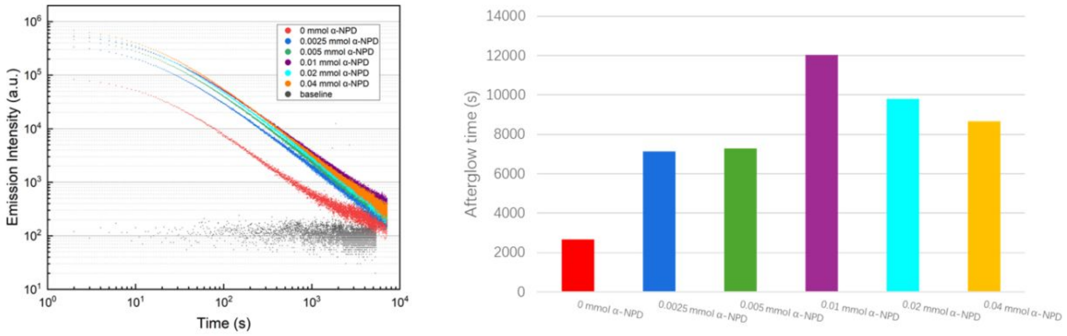

Model	ExpDec1					
Equation	y = A1*exp(-x/t1) + y0					
Plot	0 mmol NPD	0.0025 mmol NPD	0.005 mmol α-NPD	0.01 mmol α-NPD	0.02 mmol α-NPD	0.04 mmol α-NPD
y0	22116.09851 ± 997.1797	85830.82362 ± 2433.03265	119453.17392 ± 4430.42723	118828.09754 ± 2858.60864	147352.50265 ± 3682.93356	169715.28575 ± 3794.1125
A1	72686.57394 ± 705.82868	298227.07004 ± 1762.02179	402159.59606 ± 3194.23757	472313.99999 ± 2676.58101	539780.00348 ± 2758.34916	609543.73609 ± 2834.5708
t1	11.10917 ± 0.40995	10.61349 ± 0.24219	10.68774 ± 0.32735	12.72364 ± 0.24949	10.19028 ± 0.20156	10.21623 ± 0.18393
Reduced Chi-Sqr	380367.42276	2666195.05273	8623917.66202	1.20093E7	7055611.49794	7421272.5938
R-Square (COD)	0.9986	0.99942	0.99897	0.99904	0.99954	0.99962
Adj. R-Square	0.99845	0.99936	0.99886	0.99897	0.99949	0.99958

Equation	y = a + b*x					
Plot	0 mmol NPD	0.0025 mmol NPD	0.005 mmol α-NPD	0.01 mmol α-NPD	0.02 mmol α-NPD	0.04 mmol α-NPD
Weight	No Weighting					
Intercept	6.13062 ± 0.01035	6.79464 ± 0.00606	7.0072 ± 0.0056	7.03363 ± 0.00418	7.09401 ± 0.00519	7.16439 ± 0.00483
Slope	-1.12639 ± 0.00416	-1.15992 ± 0.00243	-1.1992 ± 0.00225	-1.15994 ± 0.00121	-1.20628 ± 0.00209	-1.21064 ± 0.00194
Residual Sum of Squares	0.41563	0.14213	0.12176	11.11629	0.10456	0.08767
Pearson's r	-0.99605	-0.99872	-0.99898	-0.9962	-0.99913	-0.99926
R-Square (COD)	0.99212	0.99745	0.99795	0.99242	0.99826	0.99852
Adj. R-Square	0.99211	0.99744	0.99795	0.99242	0.99826	0.99851

Figure S5. Optimization of PersL time by varying the α-NPD feed ratio in PVK exciplex system. The two tables at the bottom provide the fitted initial exponential and subsequent power law. (Excitation: 468 nm, 150 mw, 5 min; Monitored emission wavelength: 650 nm; Atmosphere environment: N_2)

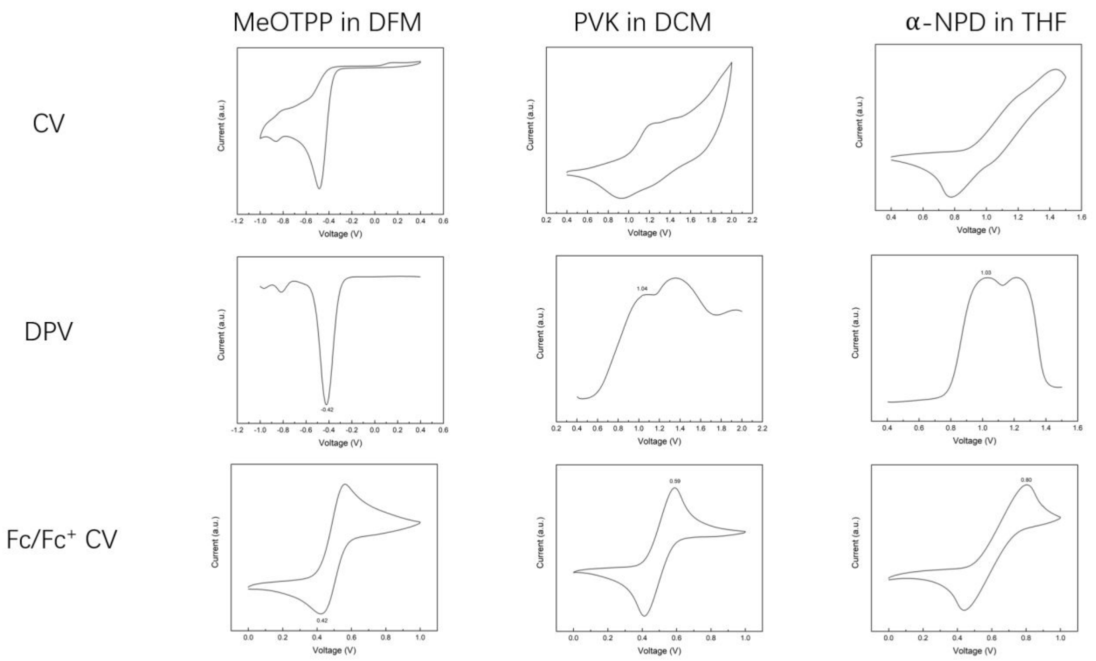

Figure S6. CV and DPV of MeOTPP, PVK, α-NPD and Fc in different solvent.

The calculations of HOMO and LUMO are based on formula $E_{HOMO/LUMO} = -(E_{ox}/E_{red} - E_{Fc/Fc^+} + 4.8)eV$.[1] For example, the LUMO of MeOTPP can be calculated from the DPV diagram, where E_{red} is -0.42 V. From CV diagram of Fc/Fc^+in DFM, E_{Fc/Fc^+}can be consider as 0.42 V. So, according to the formula, E_{LUMO} of MeOTPP is calculated to be -3.94 eV. The HOMO of PVK can be calculated from the DPV diagram, where E_{ox} is 1.04 V. From CV diagram of Fc/Fc^+in DCM, E_{Fc/Fc^+}can be consider as 0.59 V. So, according to the formula, E_{HOMO} of PVK is calculated to be -5.25 eV.

The same method can be used to calculate the HOMO of α-NPD is -5.03 eV.

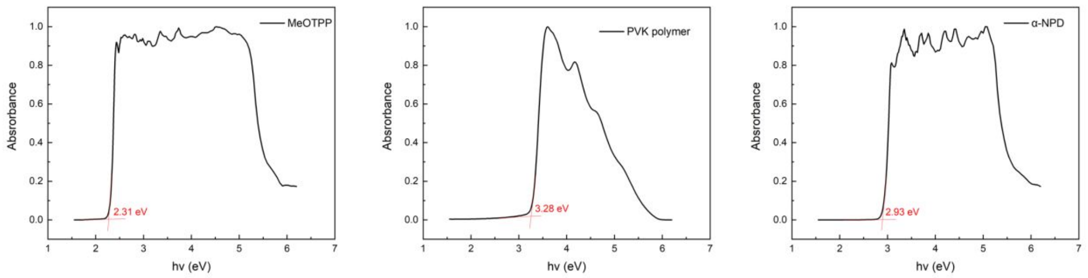

Figure S7. Absorption spectrum of MeOTPP, PVK and α-NPD.

Taking MeOTPP as an example, the optical band gap of MeOTPP is estimated to be 2.31 eV from the absorption spectrum. Combined with the LUMO level of MeOTPP (−3.94 eV) obtained from electrochemical measurements (**Figure S6)**, the HOMO level of MeOTPP can be approximated as −6.25 eV.

Using the same method, the LUMO levels of PVK and α-NPD are estimated to be −1.97 and −2.10 eV, respectively. These values are used as approximate optical estimates for energy level alignment.

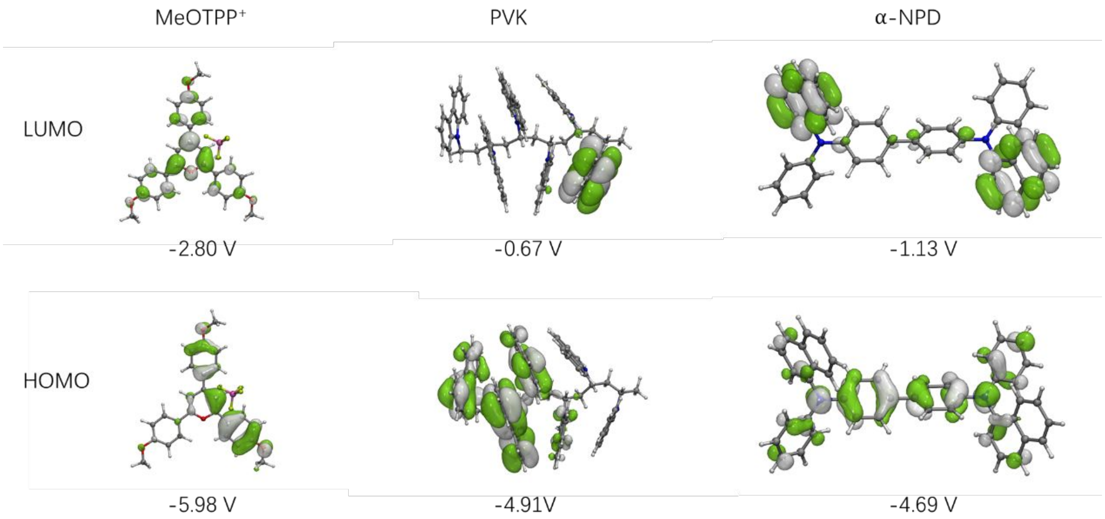

Figure S8. Natural orbitals for the ground state of MeOTPP, PVK and α-NPD.

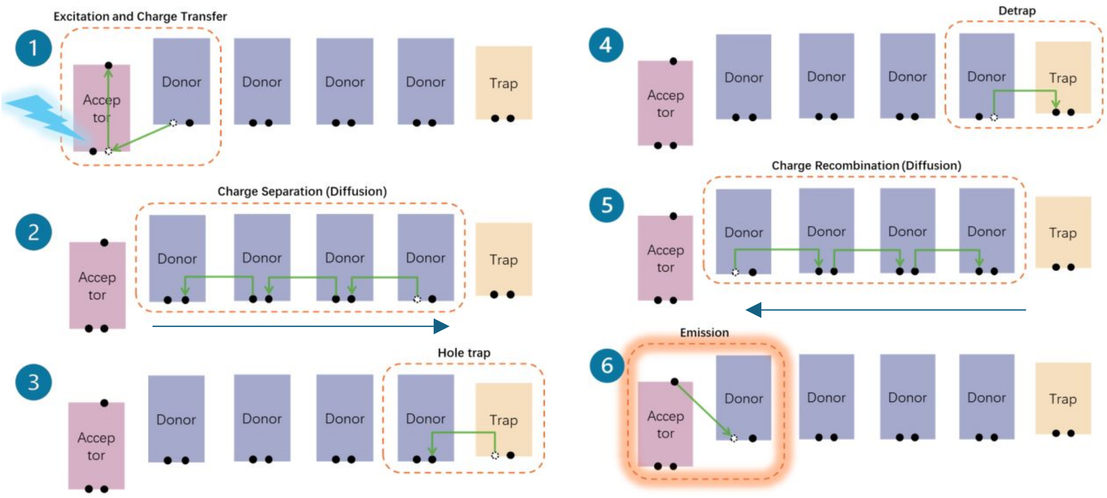

Figure S9. Specific HOMO-LUMO scheme for electron motion involving excitation, charge transfer (CT), charge separation (CS), trapping, detrapping, charge recombination (CS) and emission process in D-A-trap system.

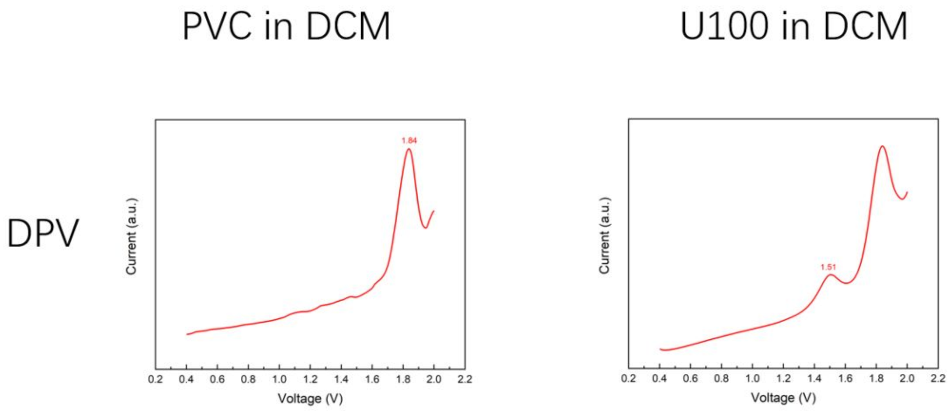

Figure S10. DPV of PVC in DCM solvent.

Using the same electrochemical method as in **Figure S6**, the HOMO levels of PVC and U100 were calculated to be −4.15 and −5.72 eV, respectively.

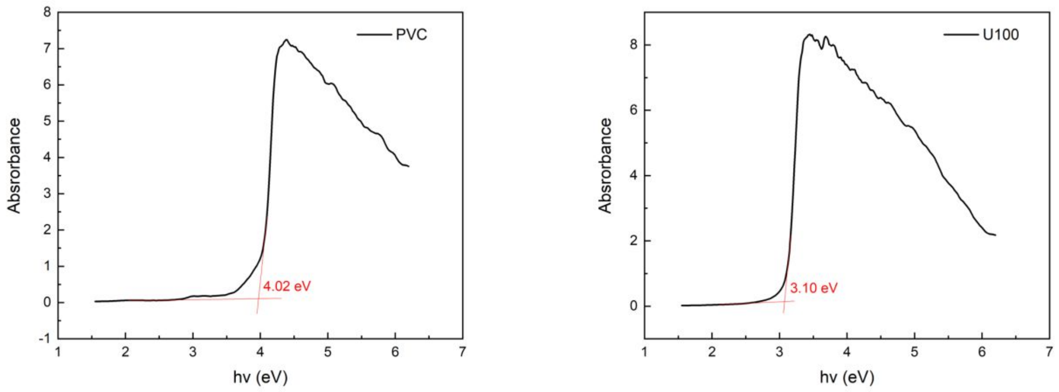

Figure S11. Absorption spectrum of PVC.

Using the same approach as in **Figure S7**, the LUMO levels of PVC and U100 are estimated to be −2.03 and −2.62 eV, respectively.

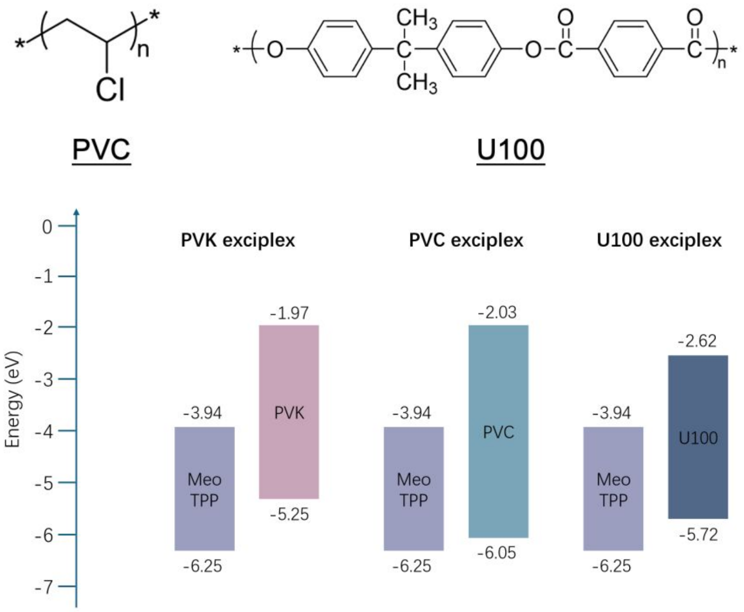

Figure S12. HOMO and LUMO energy level of PVK exciplex, PVC exciplex and U100 exciplex.

Calculated by **Figure S10 and S11.**

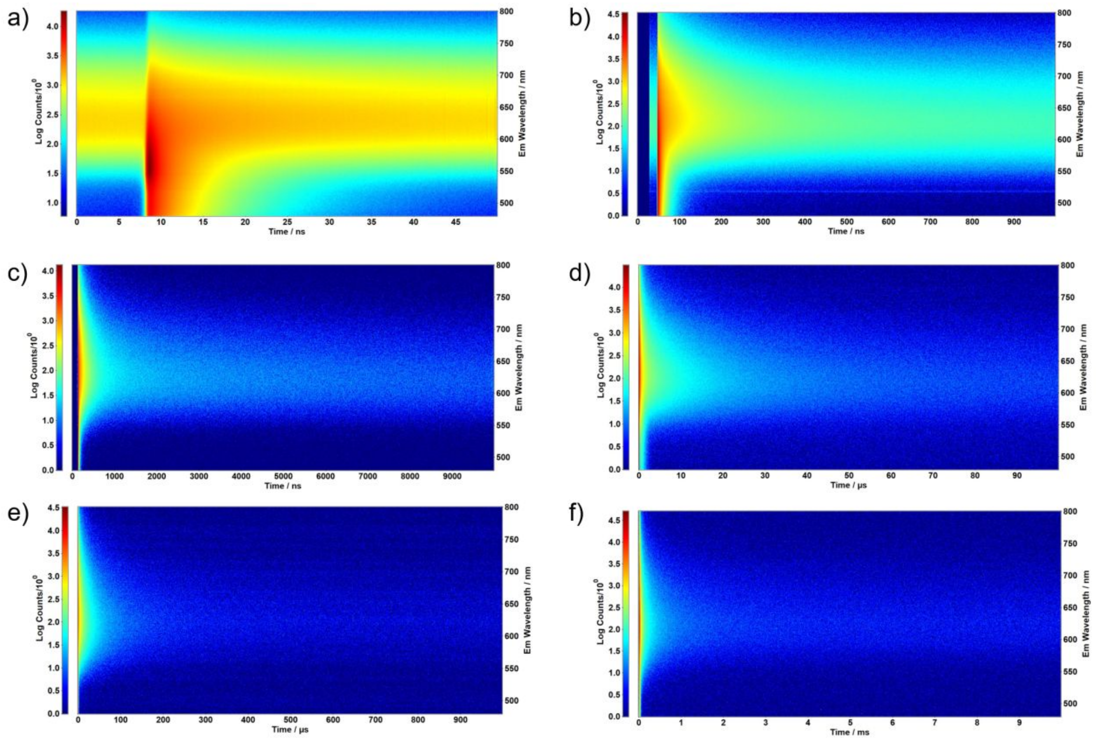

Figure S13. Time-resolved emission spectroscopy over (a) a 50 ns time range; (b) a 1 µs time range; (c) a 10 µs time range; (d) a 100 µs time range; (e) a 1 ms time range; (f) a 10 ms time range of PVK exciplex system.

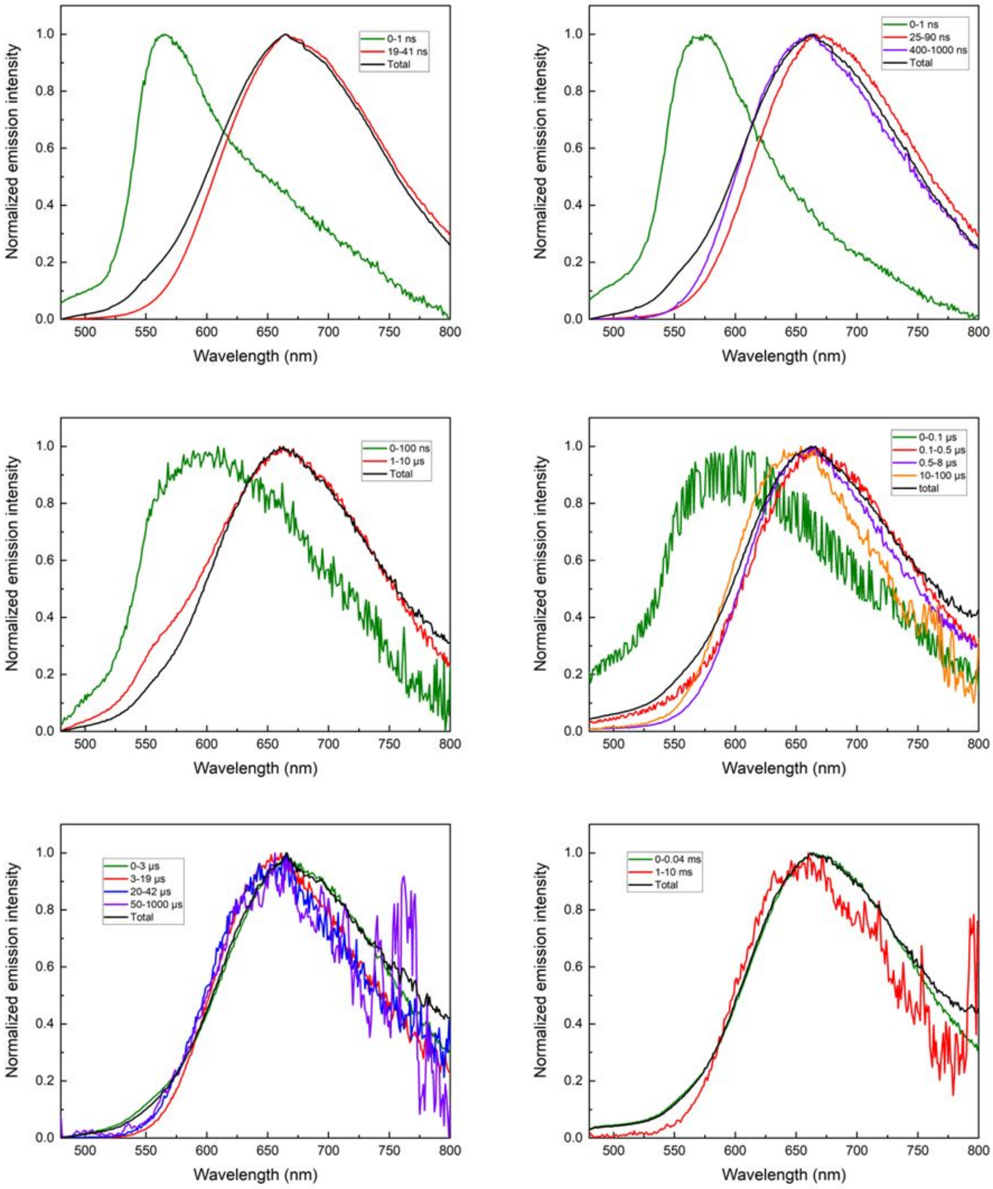

Figure S14. Time-resolved emission spectra extracted from **Figure S13** of PVK exciplex system.

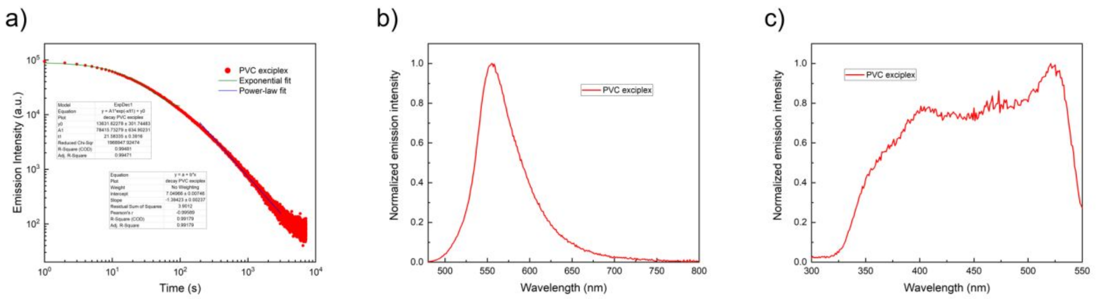

Figure S15. (a) Exponential and Power-law fitting of the decay curve for the PVC exciplex system (b) Fluorescence spectrum of PVC exciplex system under the excitation of 468 nm in ambient air; (c) Excitation spectrum of PVC exciplex system monitored at 556 nm in N_2.

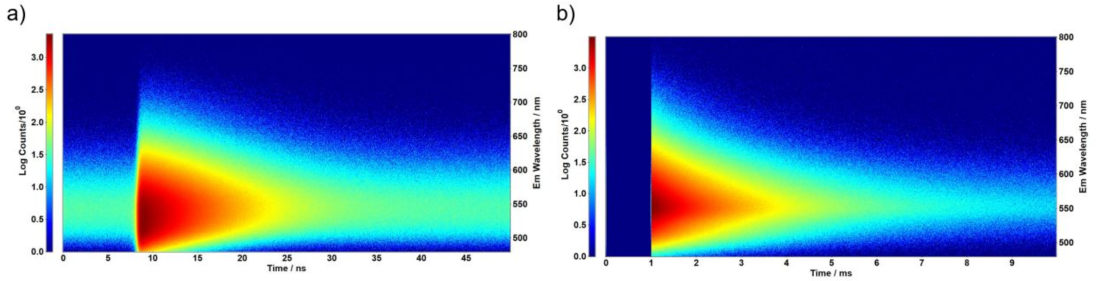

Figure S16. Time-resolved emission spectroscopy over (a) a 50 ns time range; (b) a 10 ms time range of PVC exciplex system.

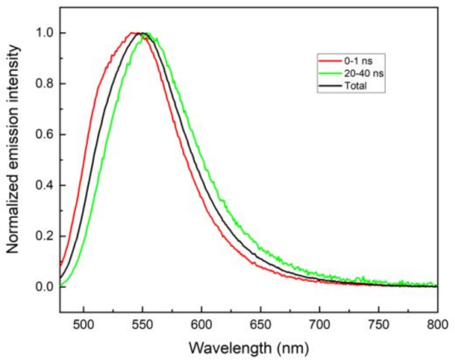

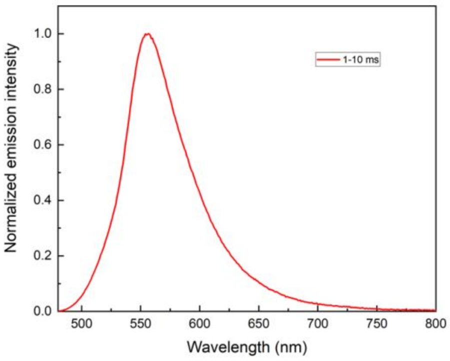

Figure S17. Time-resolved emission spectra extracted from **Figure S16** of PVC exciplex system.

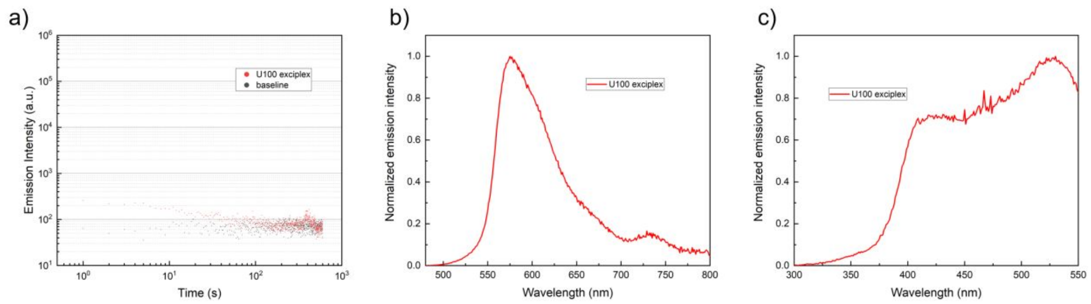

Figure S18. (a) LPL decay curves of U100 exciplex system after excitation at 468 nm for 5 min in N_2 atmosphere; (b) Fluorescence spectrum of U100 exciplex system under the excitation of 468 nm in ambient air; (c) Excitation spectrum of U100 exciplex system monitored at 575 nm in N_2.

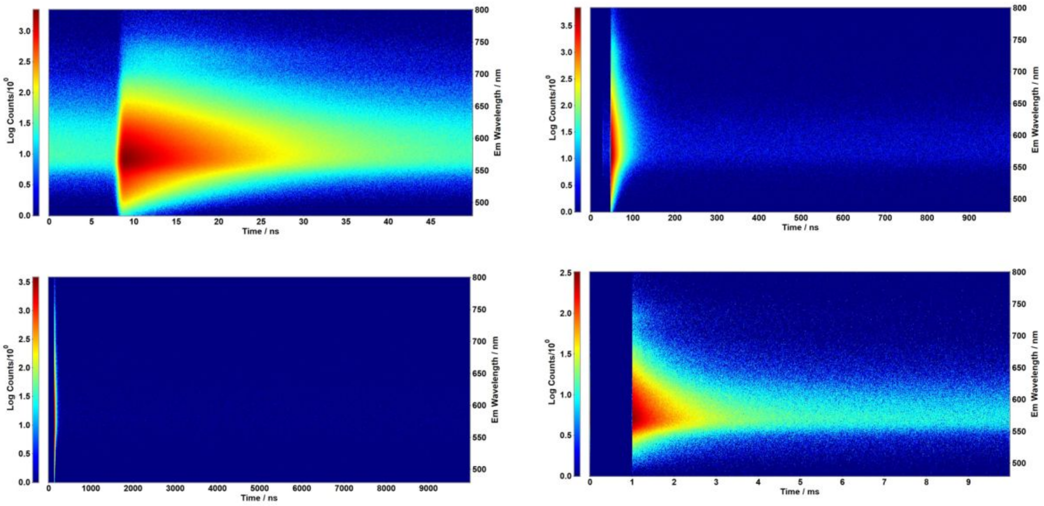

Figure S19. Time-resolved emission spectroscopy over (a) a 50 ns time range; (b) a 1 µs time range; (c) a 10 µs time range; (f) a 10 ms time range of U100 exciplex system.

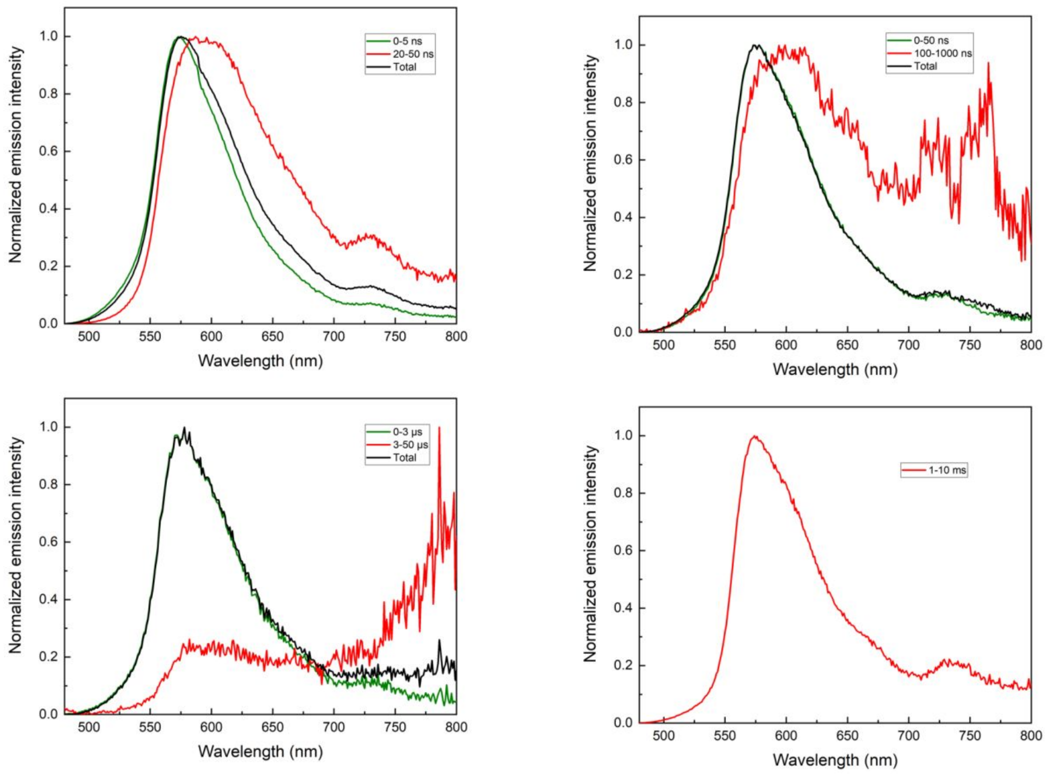

Figure S20. Time-resolved emission spectra extracted from **Figure S19** of U100 exciplex system.

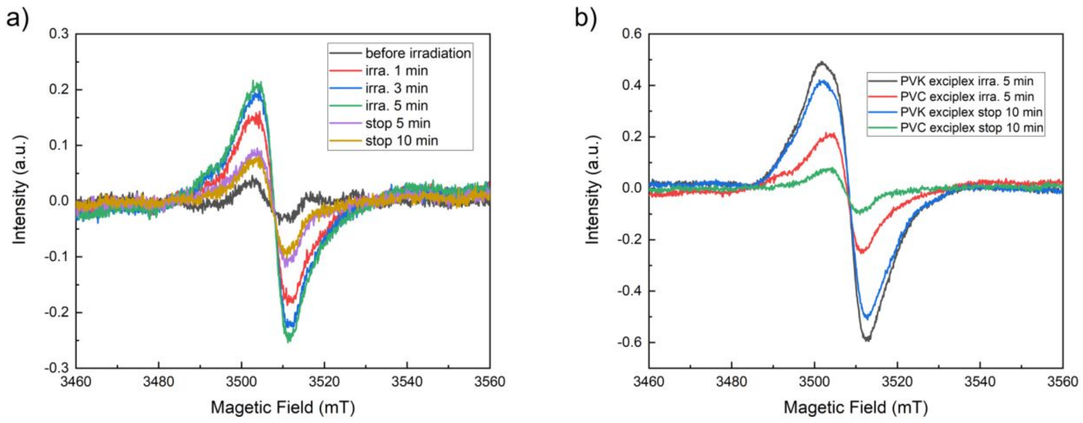

Figure S21. (a) ESR spectra of PVC exciplex system before, during and after the photo-exciation; (b) ESR spectra of PVC exciplex system and PVK system.

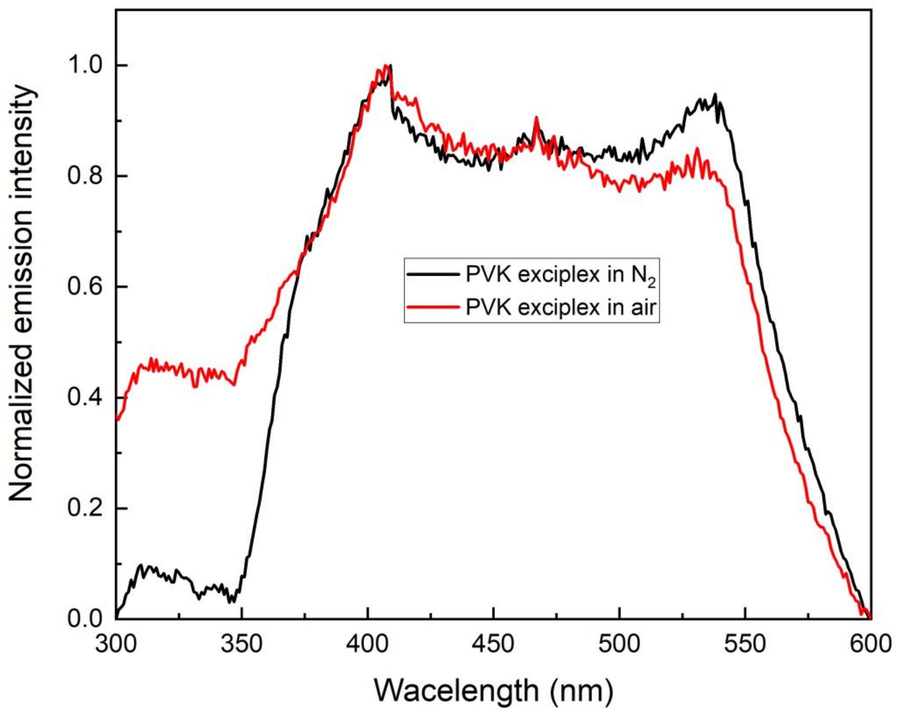

Figure S22. Excitation spectra of PVK exciplex system monitored at 650 nm in ambient air and N_2.

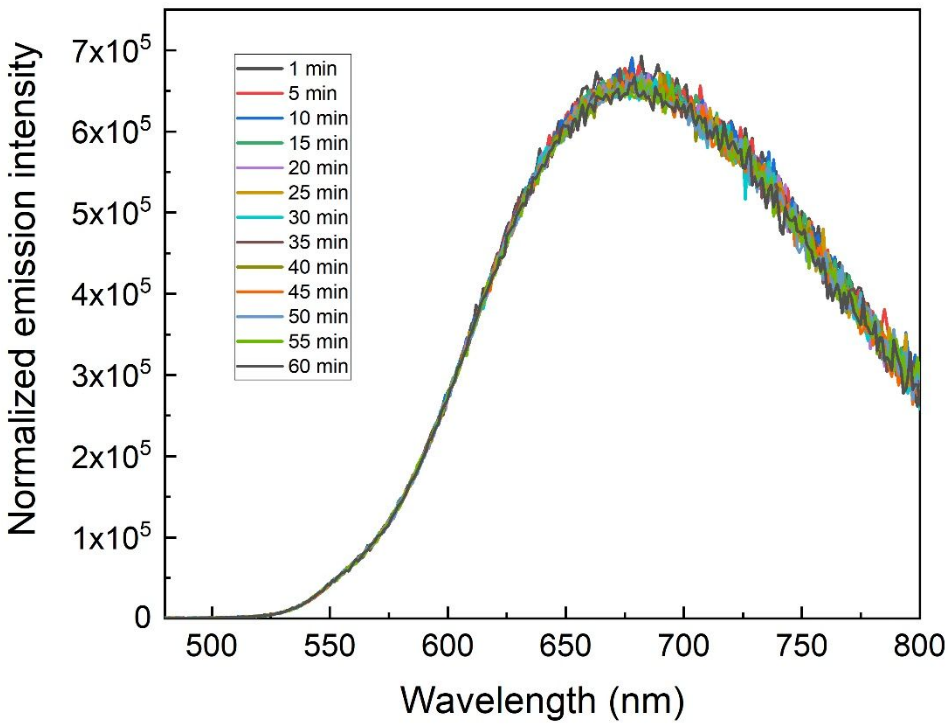

Figure S23. Emission spectra of PVK exciplex system after continuous excitation at 468 nm for different time in ambient air.

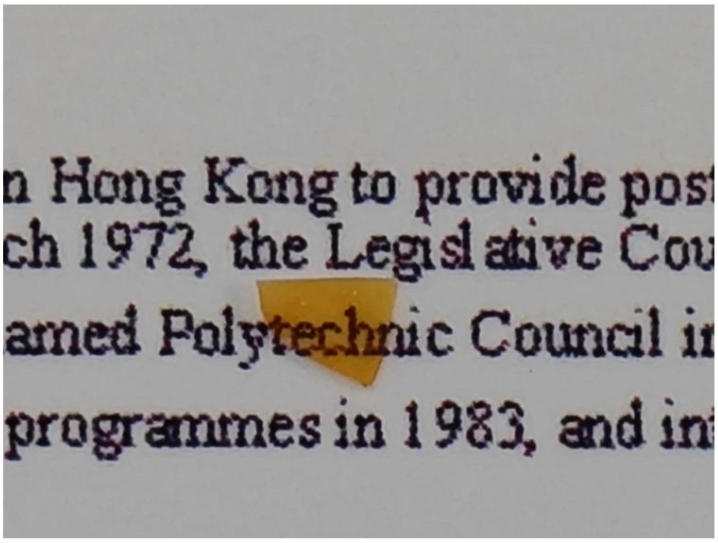

Figure S24. Photograph of synthesized PVK exciplex transparent film.

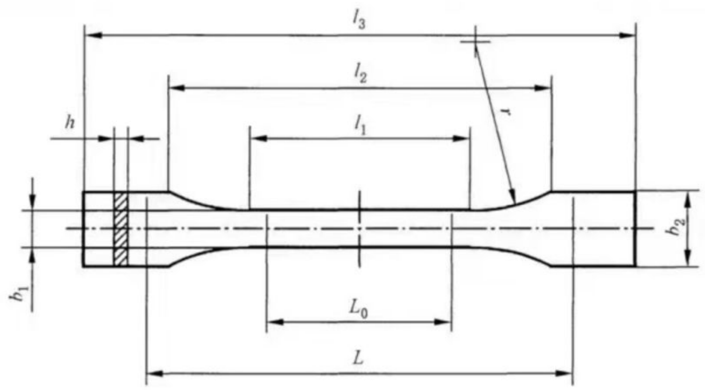

Part		Dimension (mm)
l_3	Total length	75
l_1	The length of the narrow parallel section	30
r	Radius	30
l_2	The distance of the wide parallel section	58
b_2	End Width	10
b_1	Narrow Section Width	5
h	Thickness	2
L_0	Gauge Length	25
L	Initial Distance Between Clamps	60

Figure S25. Dimensions of tensile test specimen used in this paper.

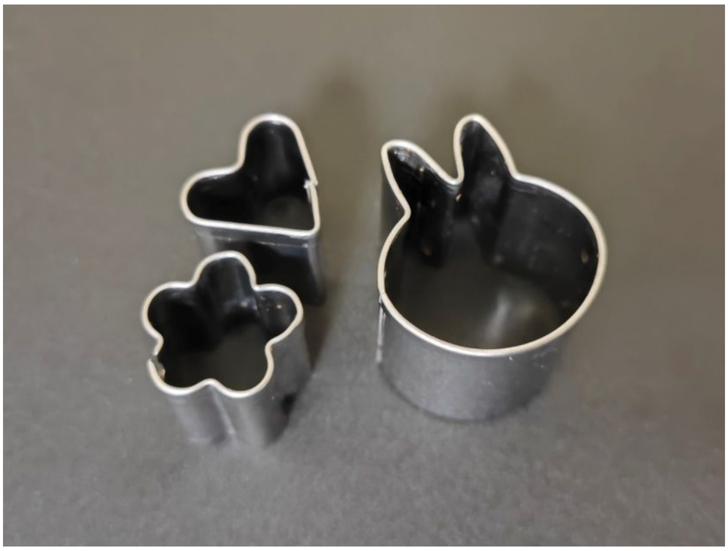

Figure S26. Different shaped die cuts.

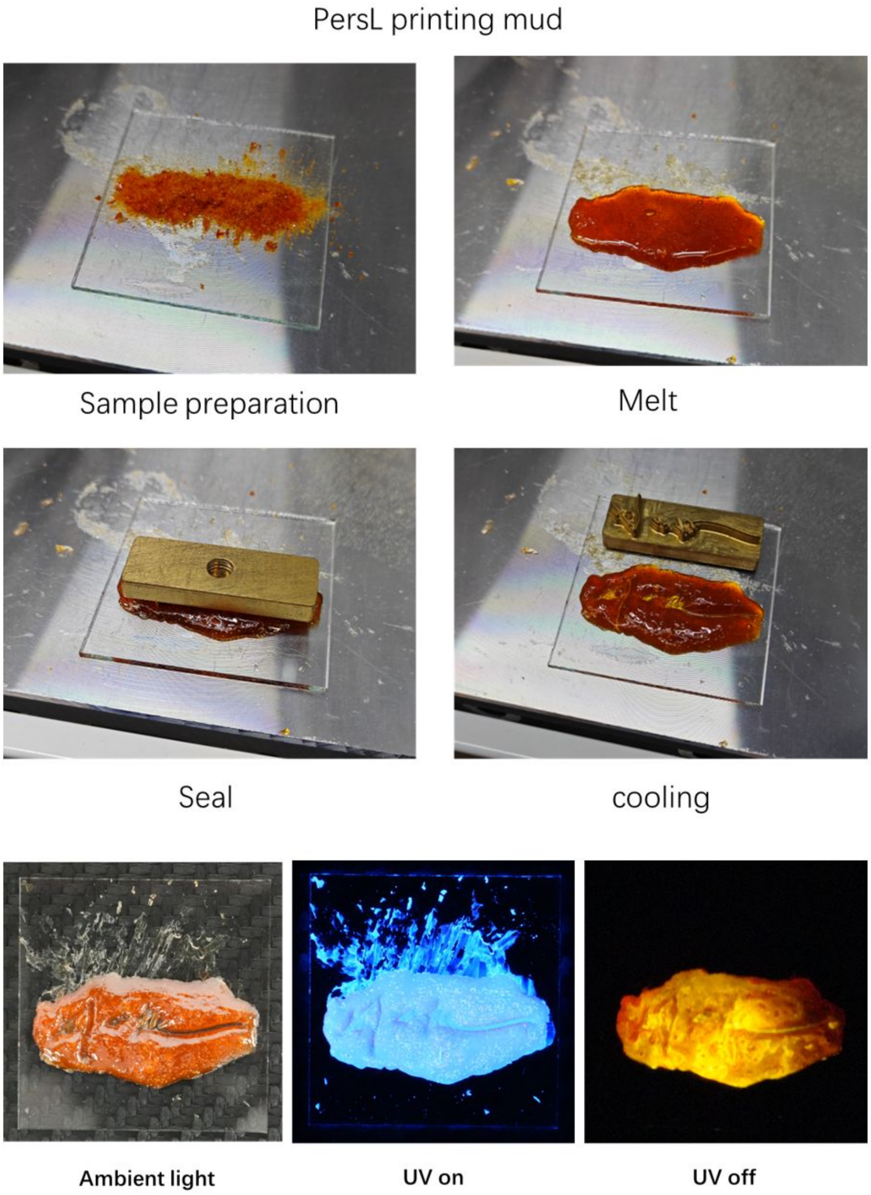

Figure S27. Stamping process by printing letters on the molten PVK exciplex with PersL properties.

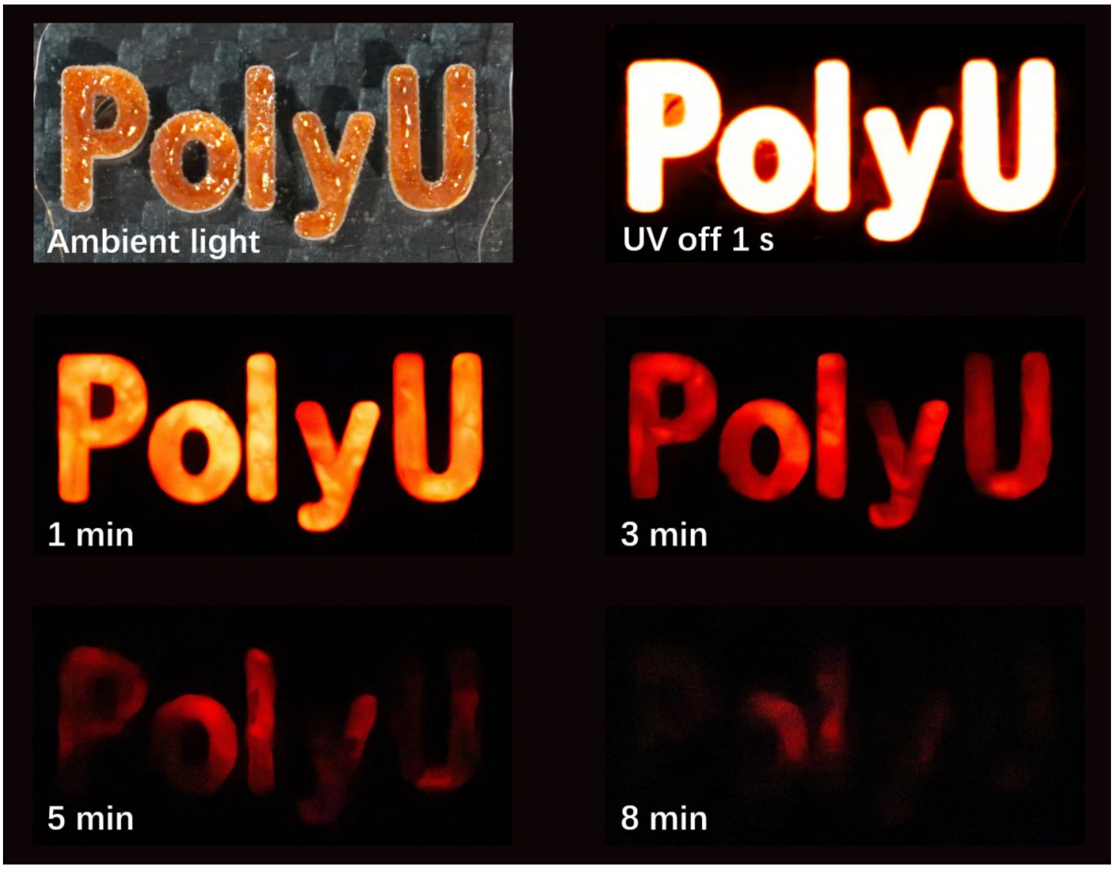

Figure S28. Letters “PolyU” molde by the PVK exciplex system.

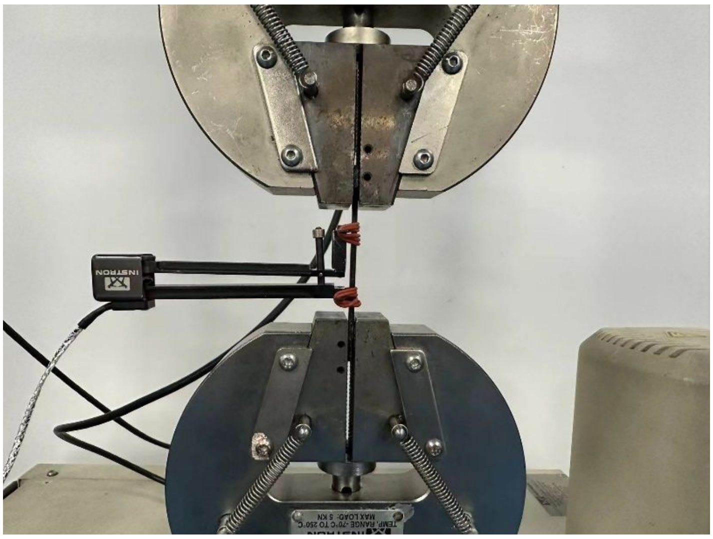

Figure S29. Device for Tensile stress-strain testing of the shaped sample PVK exciplex tensile test specimen.

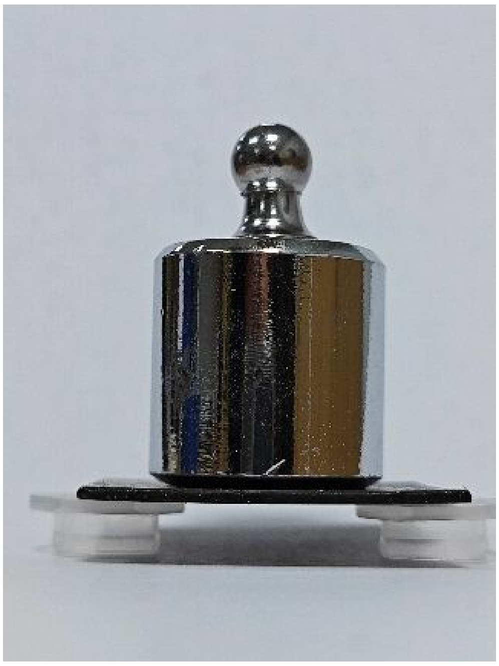

Figure S30. A 50 g weight placed on a 30 × 30 × 1 mm thin plate of the PVK exciplex system.

Table S1. Experiential fitting parameters for **Figure 5c** in 1-53 s time range.

Equation	y = A1*exp(-x/t1) + y0
y0	118828 ± 2859
A1	472314 ± 2677
t1	12.72 ± 0.25
Reduced Chi-Sqr	1.20093E7
R-Square (COD)	0.99904
Adj. R-Square	0.99897

Table S2. Power law fitting parameters for **Figure 5c** in 57-7200 s time range.

Equation	y = a + b*x
a	7.034 ± 0.004
b	-1.160 ± 0.001
Residual Sum of Squares	11.11629
Pearson's r	-0.9962
R-Square (COD)	0.99242
Adj. R-Square	0.99242

Table S3. Experiential fitting parameters for **Figure 5f** in 1-99 s time range.

Equation	y = A1*exp(-x/t1) + y0
y0	13632 ± 302
A1	78416 ± 635
t1	21.58 ± 0.39
Reduced Chi-Sqr	1968947.92474
R-Square (COD)	0.99481
Adj. R-Square	0.99471

Table S4. Power law fitting parameters for **Figure 5f** in 195-3055 s time range.

Equation	y = a + b*x
a	7.050 ± 0.007
b	-1.394 ± 0.002
Residual Sum of Squares	3.9012
Pearson's r	-0.99589
R-Square (COD)	0.99179
Adj. R-Square	0.99179

Table S5. Comparison of the calculated Young's modulus of the PVK exciplex system with that of bakelite and aluminum-nickel alloys.

	Young’s modulus
PVK exciplex	1.95
Rubber	0.01
Bakelite	1.96-2.94
Aluminum-nickel alloys	2.70

Table S6. Comparison of the Shore hardness HD of the PVK exciplex system with that of solid truck tires and hard hat.

	Hardness HD
PVK exciplex	75.3
Solid truck tires	50.0
Hard hat	75.0

References

[1] Liu, Y., Liu, M. S. & Jen, A. K. Y. Synthesis and characterization of a novel and highly efficient light-emitting polymer. *Acta Polymerica* **50**, 105–108 (1999)
